# Author Correction: Detection of aberrant splicing events in RNA-seq data using FRASER

**DOI:** 10.1038/s41467-022-31242-2

**Published:** 2022-06-16

**Authors:** Christian Mertes, Ines F. Scheller, Vicente A. Yépez, Muhammed H. Çelik, Yingjiqiong Liang, Laura S. Kremer, Mirjana Gusic, Holger Prokisch, Julien Gagneur

**Affiliations:** 1 Department of Informatics, Technical University of Munich, Garching, Germany; 2 Institute of Computational Biology, Helmholtz Zentrum München, Neuherberg, Germany; 3 Quantitative Biosciences Munich, Gene Center, Ludwig-Maximilians Universität München, Munich, Germany; 4 Institute of Human Genetics, Helmholtz Zentrum München, Neuherberg, Germany; 5 Institute of Human Genetics, Klinikum rechts der Isar, Technical University of Munich, Munich, Germany

**Keywords:** Computational models, Disease genetics, RNA splicing, RNA sequencing

Correction to: *Nature Communications* 10.1038/s41467-020-20573-7, published online 22 January 2021.

In the original version of this Article, it was incorrectly stated that the GTEx dataset version V7 aligned with STAR was used. Throughout, the GTEx dataset version V6p aligned with TopHat was used. In addition, it was stated incorrectly that we filtered out splice sites and introns with zero coverage in 95% of the sample. Instead, we kept only splice sites and introns with at least one read coverage in 95% of the samples. These errors do not affect any of the results or conclusions of the study. Corrections to the text are listed below.

The original version of this Article contained an error in the first sentence of the second paragraph of the Results section, which incorrectly read ‘To establish FRASER, we considered the GTEx project dataset (V7)^24^.’ The correct version states ‘V6p’ in place of ‘V7’.

The first sentence of the Methods subsection ‘Datasets’ originally incorrectly read ‘7842 RNA-seq samples from 48 tissues of 543 assumed healthy individuals of the Genotype-Tissue Expression Project V7^24^ (hereafter the GTEx dataset).’ The correct version states ‘V6p’ in place of ‘V7’.

The fifth and sixth sentences of the Methods subsection ‘Datasets’ originally incorrectly read ‘For GTEx, we obtained the BAM files from dbGaP (phs000424.v7.p2), which were already aligned with STAR (version 2.4.2a). The GTEx consortia used the same parameters, with the exception of mapping against the GRCh37 genome assembly^49^ based on the GENCODE v19 annotation^28^.’ The correct version replaces these sentences with ‘For GTEx, we obtained the BAM files from dbGaP (phs000424.v6.p1), which were already aligned by the GTEx consortium with TopHat (version v1.4) against the GRCh37 genome assembly^49^ based on the GENCODE v19 annotation^28^.’

The last sentence of the Methods subsection 'Read counting and splicing metrics' originally incorrectly read 'Further, we filtered out splice sites and introns where more than 95% of the samples had zero coverage.' The correct version replaces this sentence with 'Further, we kept only splice sites and introns with at least one read coverage in 95% of the samples.’

The first sentence of the Methods subsection ‘Enrichment analysis’ originally incorrectly read ‘from the GTEx whole-genome sequencing genetic variant data (V7)^24^.’ The correct version states ‘V6p’ in place of ‘V7’.

The first sentence of the Data Availability statement originally incorrectly read ‘The GTEx dataset is available through dbGaP (accession number: phs000424.v7.p2).’ The correct version states ‘phs000424.v6.p1’ in place of ‘phs000424.v7.p2’.

The final sentence of the Acknowledgements section originally incorrectly read ‘under accession number dbGaP:phs000424.v7.p2.’ The correct version states ‘phs000424.v6.p1’ in place of ‘phs000424.v7.p2’.

These errors have been corrected in both the PDF and the HTML versions of the Article.

