# Supplementary material for: Detection of aberrant splicing events in RNA-seq data using FRASER
Source: Nat Commun. 2021 Jan 22;12:529. doi: 10.1038/s41467-020-20573-7 (PMC7822922; doi:10.1038/s41467-020-20573-7)
Supplement: Supplementary file 1 — Supplementary Information [file 41467_2020_20573_MOESM1_ESM.pdf]

Detection of aberrant splicing events in RNA-seq data using FRASER

Christian Mertes[1,*] Ines Scheller[1,2,*] Vicente A. Yepez[1,3]
Muhammed H. Celik[1] Yingjiqiong Liang[1] Laura S. Kremer[4,5]
Mirjana Gusic[4,5] Holger Prokisch[4,5] Julien Gagneur[1,2]

[1]Department of Informatics, Technical University of Munich, Garching, Germany
[2]Institute of Computational Biology, Helmholtz Zentrum München, Neuherberg, Germany
[3]Quantitative Biosciences Munich, Gene Center, Ludwig-Maximilians Universität München, Munich, Germany
[4]Institute of Human Genetics, Helmholtz Zentrum München, Neuherberg, Germany
[5]Institute of Human Genetics, Klinikum rechts der Isar, Technical University of Munich, Munich, Germany

[*]Contributed equally to this work

Supplementary Figures

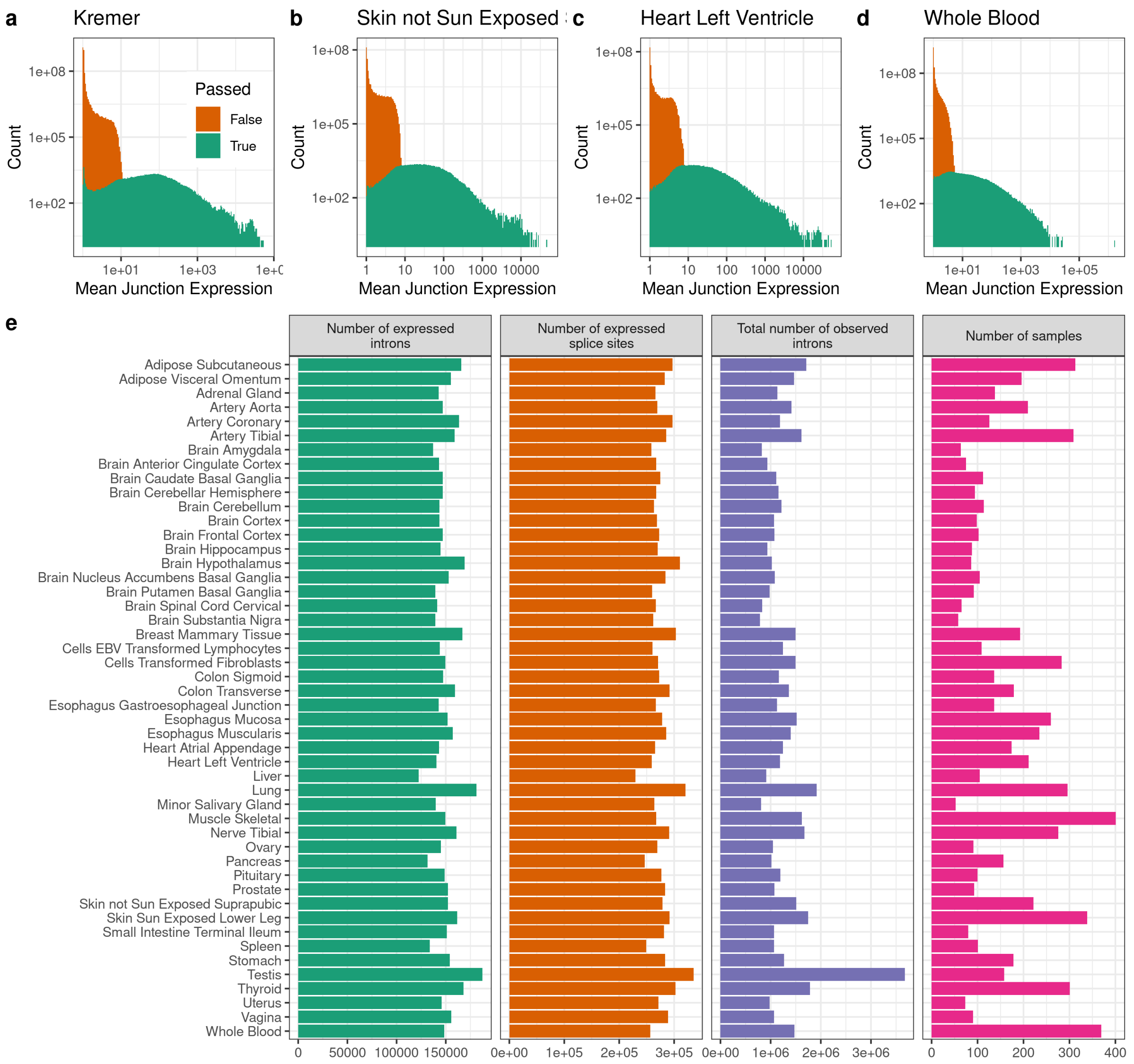

Figure S1: **Filtering of introns.** **(a)** Histogram of the raw intron coverage per sample-intron pair for the Kremer data set grouped according to the intron filter status. Green indicates that the intron passed the filter and orange indicates that the intron was filtered out. **(b-d)** Same as (a), but for different tissues in the GTEx data set. **(e)** Barplots of the number of introns passed the filtering, splice sites passed the filtering, observed introns, and samples per tissue within the GTEx data set used in this study.

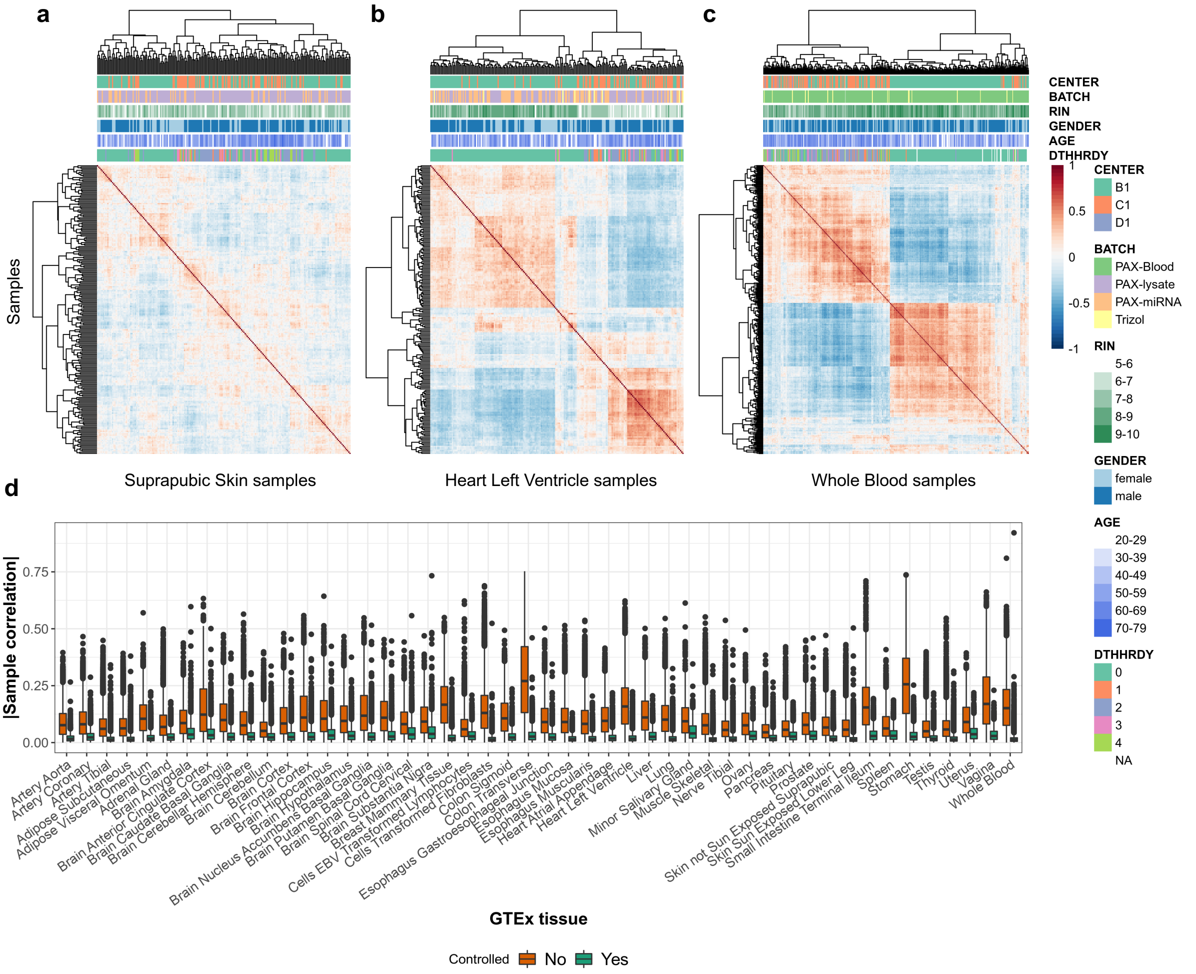

Figure S2: **Tissue-specific correlation structure for ψ_3. (a-c)** Intron-centered and logit-transformed ψ_3 of the 10,000 most variable introns clustered by samples (columns and rows) for three representative GTEx tissues: suprapubic skin (a, n=222), left ventricle heart (b, n=211), and whole blood (c, n=369). Red and blue depict relative high and low intron usage, respectively. Colored horizontal tracks display sequencing center, batch, RNA integrity number (RIN), gender, age, and cause of death (DTHHRDY, Hardy scale classification) of the samples. **(d)** Boxplots of absolute values of between-sample correlations of row-centered logit-transformed ψ_3 for 48 GTEx tissues before (orange) and after (green) correction for the latent space (n = number of sample-pairs per GTEx tissue, between 52 and 401 samples per tissue, for more details see Supplementary Figure S1E). The intron-centered ψ_3 values were clipped to the $[0.01, 0.99]$ interval before logit-transformation. The data in (d) are represented as boxplots in which the middle line indicates the median, the bounds of the box indicate the first and third quartiles, and the whiskers indicate +/- 1.5 x IQR from the third and first quartile, respectively (IQR = interquartile range). Outlying data points are shown as dots.

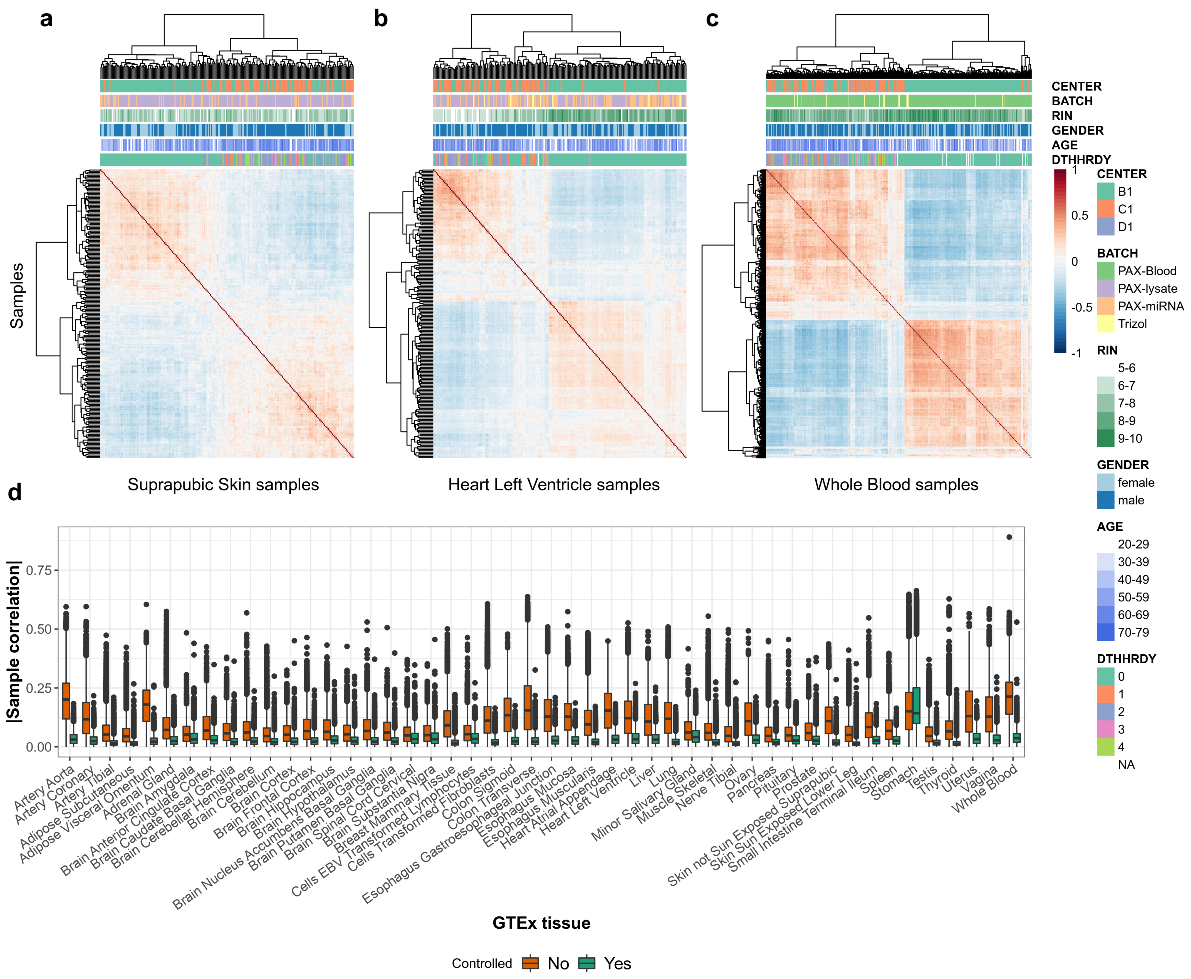

Figure S3: **Tissue-specific correlation structure for θ. (a-c)** Splice-site-centered and logit-transformed θ of the 10,000 most variable splice sites clustered by samples (columns and rows) for three representative GTEx tissues: suprapubic skin (a, n=222), left ventricle heart (b, n=211), and whole blood (c, n=369). Red and blue relative depict high and low intron usage, respectively. Colored horizontal tracks display sequencing center, batch, RNA integrity number (RIN), gender, age, and cause of death (DTHHRDY, Hardy scale classification) of the samples. **(d)** Boxplots of absolute values of between-sample correlations of row-centered logit-transformed θ for 48 GTEx tissues before (orange) and after (green) correction for the latent space (n = number of sample-pairs per GTEX tissue, between 52 and 401 samples per tissue, for more details see Supplementary Figure S1E). The splice-site-centered θ values were clipped to the $[0.01, 0.99]$ interval before logit-transformation. The data in (d) are represented as boxplots in which the middle line indicates the median, the bounds of the box indicate the first and third quartiles and the whiskers indicate +/- 1.5 x IQR from the third and first quartile, respectively (IQR = interquartile range). Outlying data points are shown as dots.

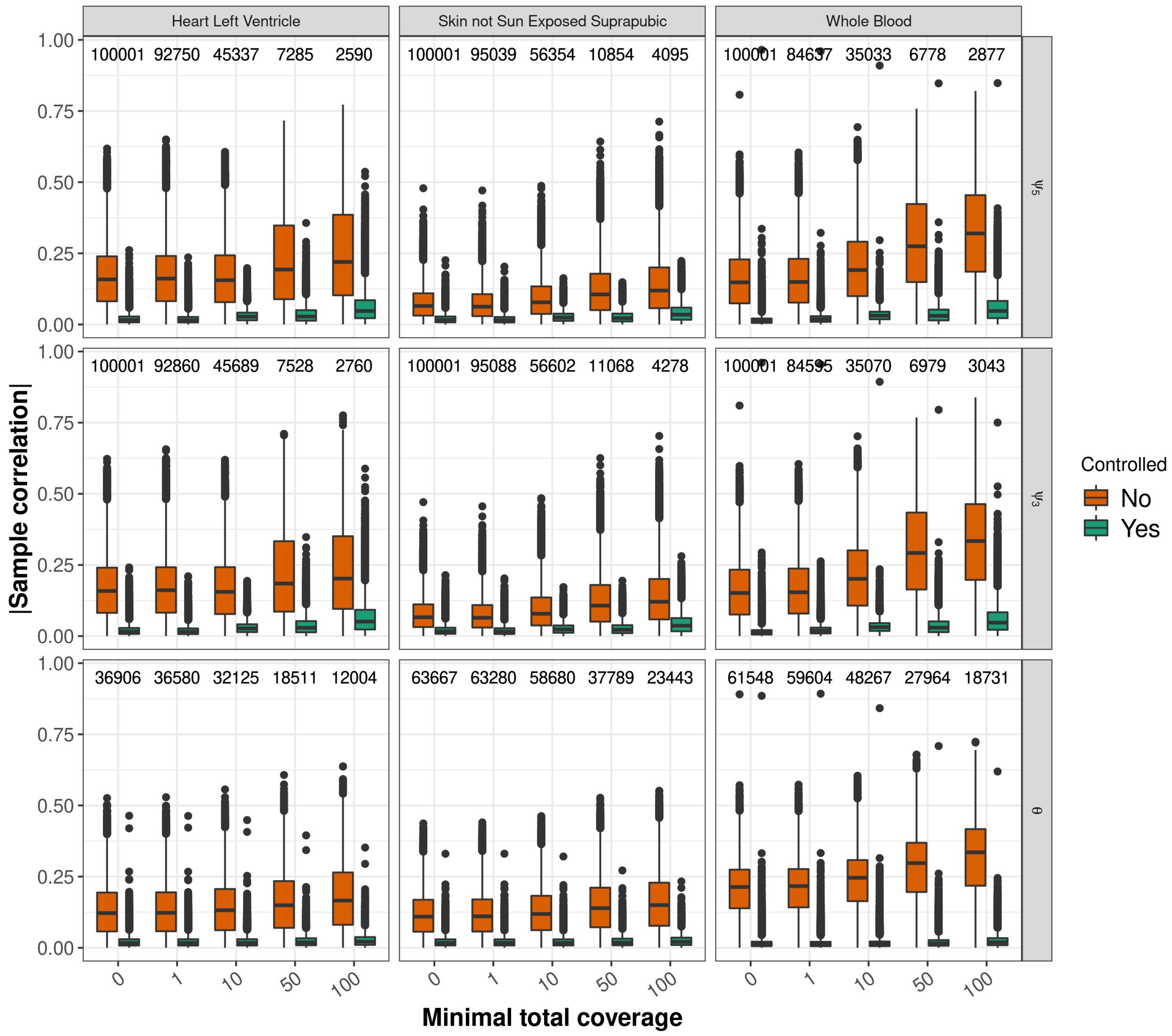

Figure S4: **Effect of total expression on sample correlation structure.** Boxplots of absolute values of pairwise between-sample correlations of intron-centered logit-transformed splicing metrics (rows) for three representative GTEx tissues (columns) before (orange) and after (green) correction for the latent space (n = 211 samples for Heart Left Ventricle, n = 222 samples for Skin and n = 369 samples for Whole Blood). The x-axis indicates the minimal total coverage (N) for an intron in both samples to be considered in the calculation of the pairwise sample correlation. The splicing metrics were clipped to the $[0.01, 0.99]$ interval before logit-transformation. The number above each boxplot indicates the mean number of introns (or splice sites for θ) that could be used for calcuating the sample correlation for each sample-pair given the corresponding cutoff on the minimal total coverage. The data in this figure is represented as boxplots in which the middle line indicates the median, the bounds of the box indicate the first and third quartiles and the whiskers indicate +/- 1.5 x IQR from the third and first quartile, respectively (IQR = interquartile range). Outlying data points are shown as dots.

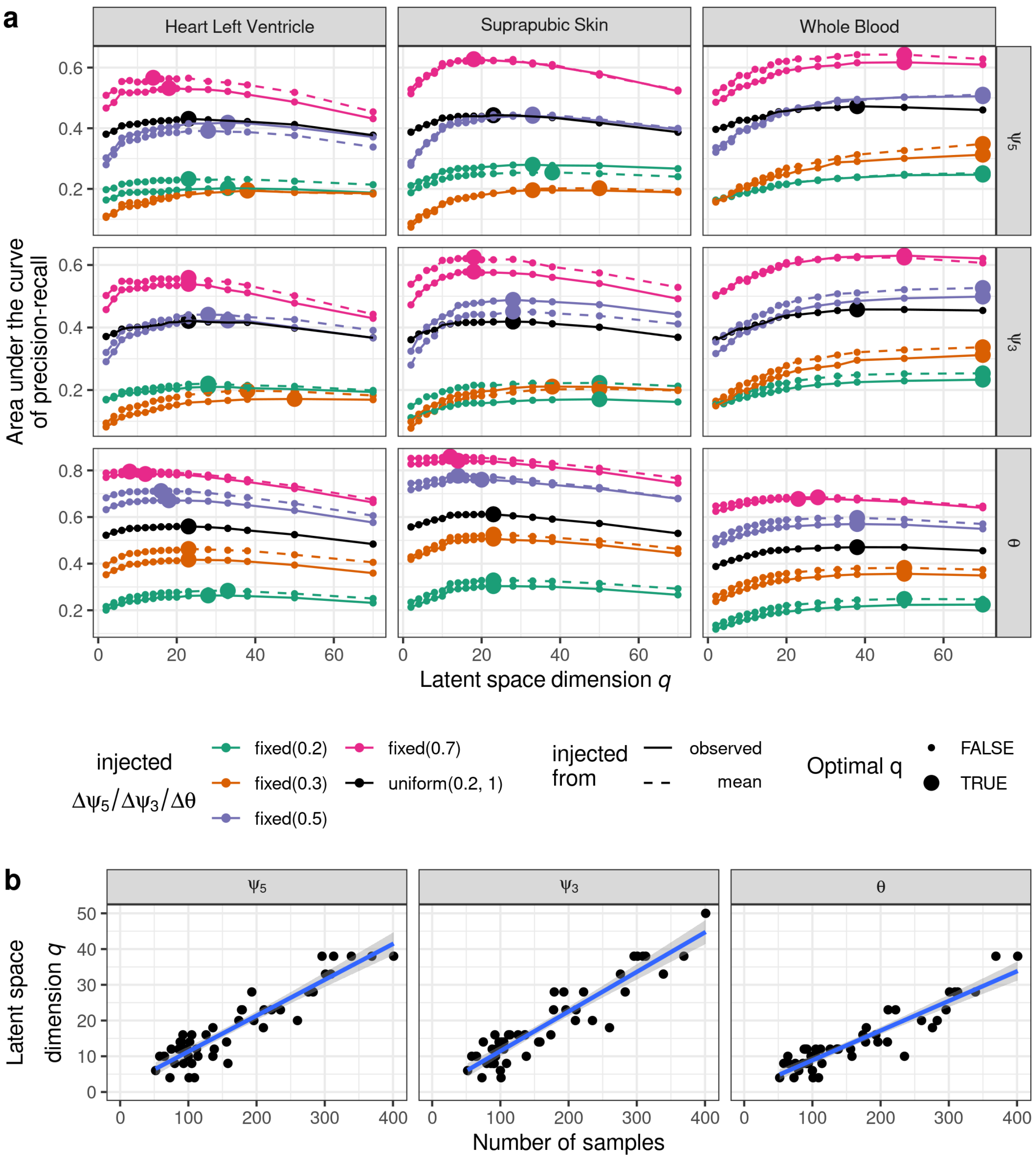

Figure S5: **Finding the optimal latent space dimension** q. **(a)** Area under the precision-recall curve for recalling artificially injected outliers (y-axis) against latent space dimension q (x-axis) stratified by splicing metrics (rows) and three representative GTEx datasets (columns). Simulated outliers are generated using different scenarios: By shifting the splicing metrics away from its observed value (plain) or from its average across samples (dashed) and with shift of various amplitudes: 0.2 (green), 0.3 (orange), 0.5 (purple) or 0.7 (pink) as well as with amplitudes drawn uniformly in $[0.2, 1]$ (black). For each scenario, the optimal latent space dimension q is marked with a thicker dot. **(b)** For each of the 48 GTEx tissues, the number of samples are plotted against the estimated latent space dimension. The data is stratified by the splicing metrics (columns). The blue line represents a linear regression fit and the gray band around it defines the 95% confidence interval of the fit.

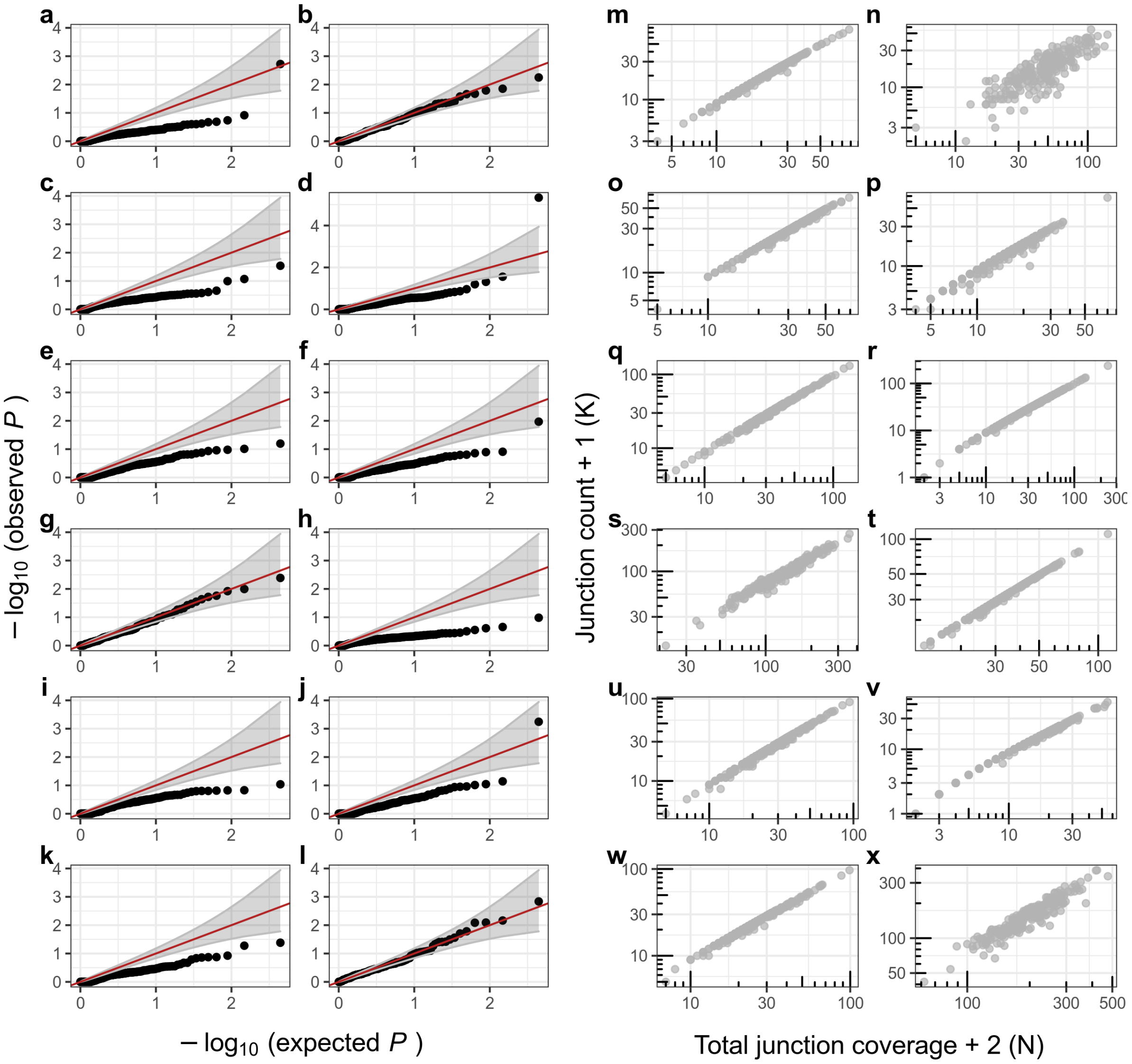

Figure S6: **Quantile-quantile plots and the corresponding count ratios. (a-l)** Quantile-quantile plots of randomly chosen introns based on the ψ_5 metric. Under the null hypothesis, the data are expected to lie along the diagonal (red, 95% confidence bands in gray). **(m-x)** Expression plots of the number of split reads (K) over the total coverage (N) of the given donor site. The q-q plots (a-l) correspond to the respective expression plot (m-x). The data is based on the suprapubic skin GTEx tissue (n=222).

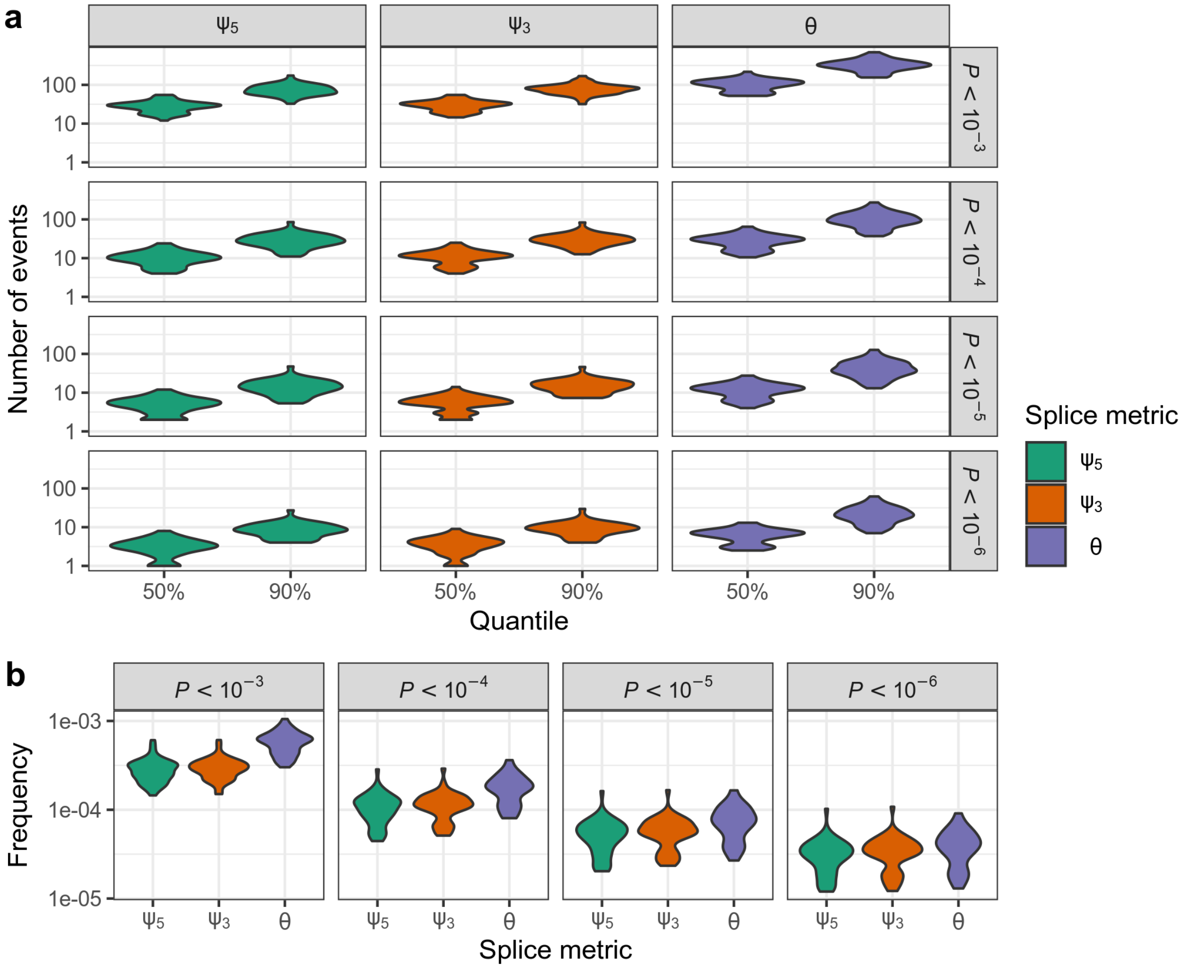

Figure S7: **Distribution of extreme beta-binomial *P* values across GTEx tissues.** **(a)** The distribution of the number of events (y-axis) having a more significant P value than a given cutoff (rows) is plotted against the quantiles across samples within a tissue (x-axis, median and 90%). The data is stratified by the different splicing metrics (columns, with green, orange, and purple for ψ_5, ψ_3, and θ, respectively). Each distribution is based on the 48 GTEx tissues. **(b)** Same as (a) but the frequency (y-axis) of P values being smaller than a given cutoff (columns) across a tissue is plotted per splicing metrics (x-axis). In panels (a - b), the P values underlying the distributions were calculated two-sided with the beta-binomial distribution (Methods).

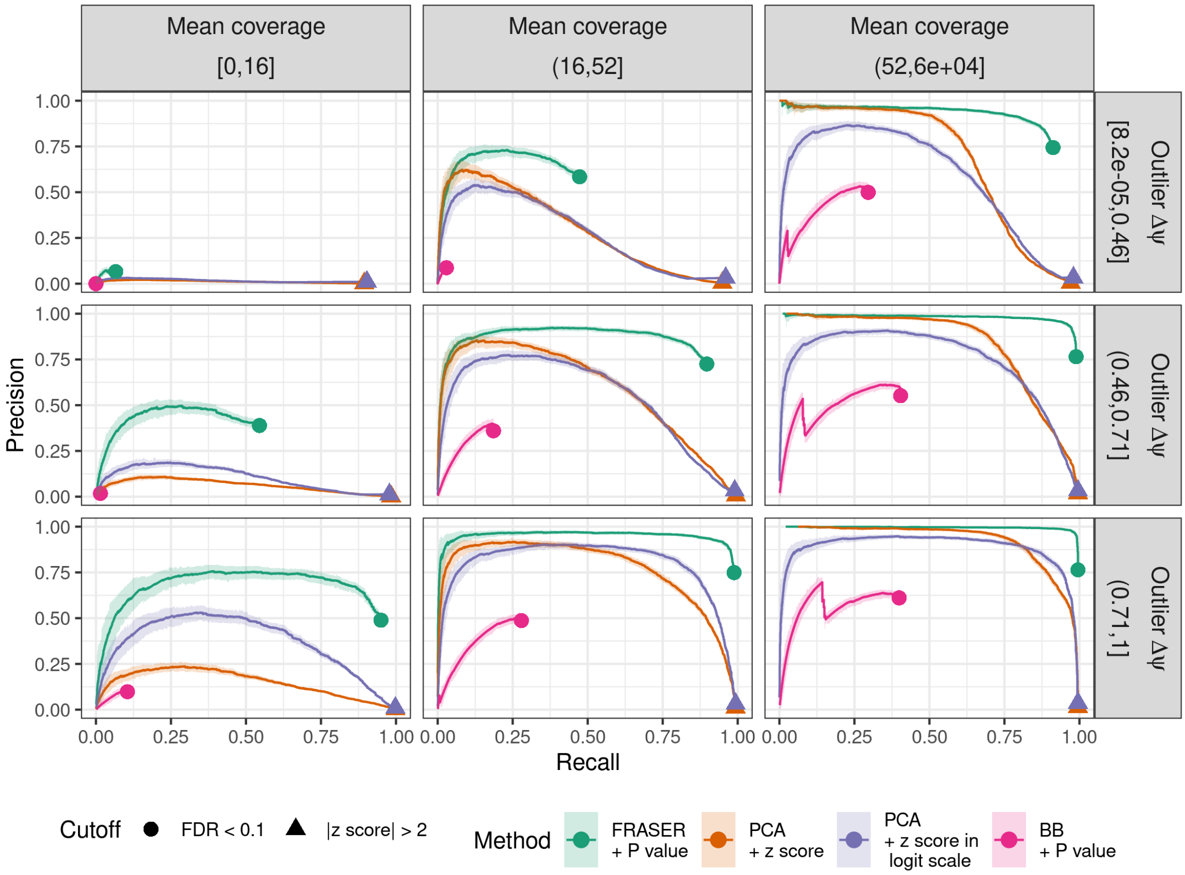

Figure S8: **Splicing outlier detection benchmark in GTEx for ψ_5.** The proportion of simulated outliers among reported outliers (precision) plotted against the proportion of reported simulated outliers among all simulated outliers (recall) for increasing P values (FRASER, green; beta binomial, pink) or decreasing absolute PCA-based z scores (natural scale, orange, Supplementary Note 2; logit scale, purple). Moreover, all events with $|\Delta\psi_5| < 0.1$ are ranked last. The data are stratified by the mean coverage of the intron (columns) and by the injected absolute ψ_5 value (rows). The cutoffs for each method are marked (FDR < 0.1, circle; absolute z score > 2, triangle). The darker lines mark the precision-recall curves computed for the full dataset while the light ribbons around the curves indicate 95% confidence bands estimated by bootstrapping. Abbreviations: BB, beta-binomial; PCA, principal component analysis.

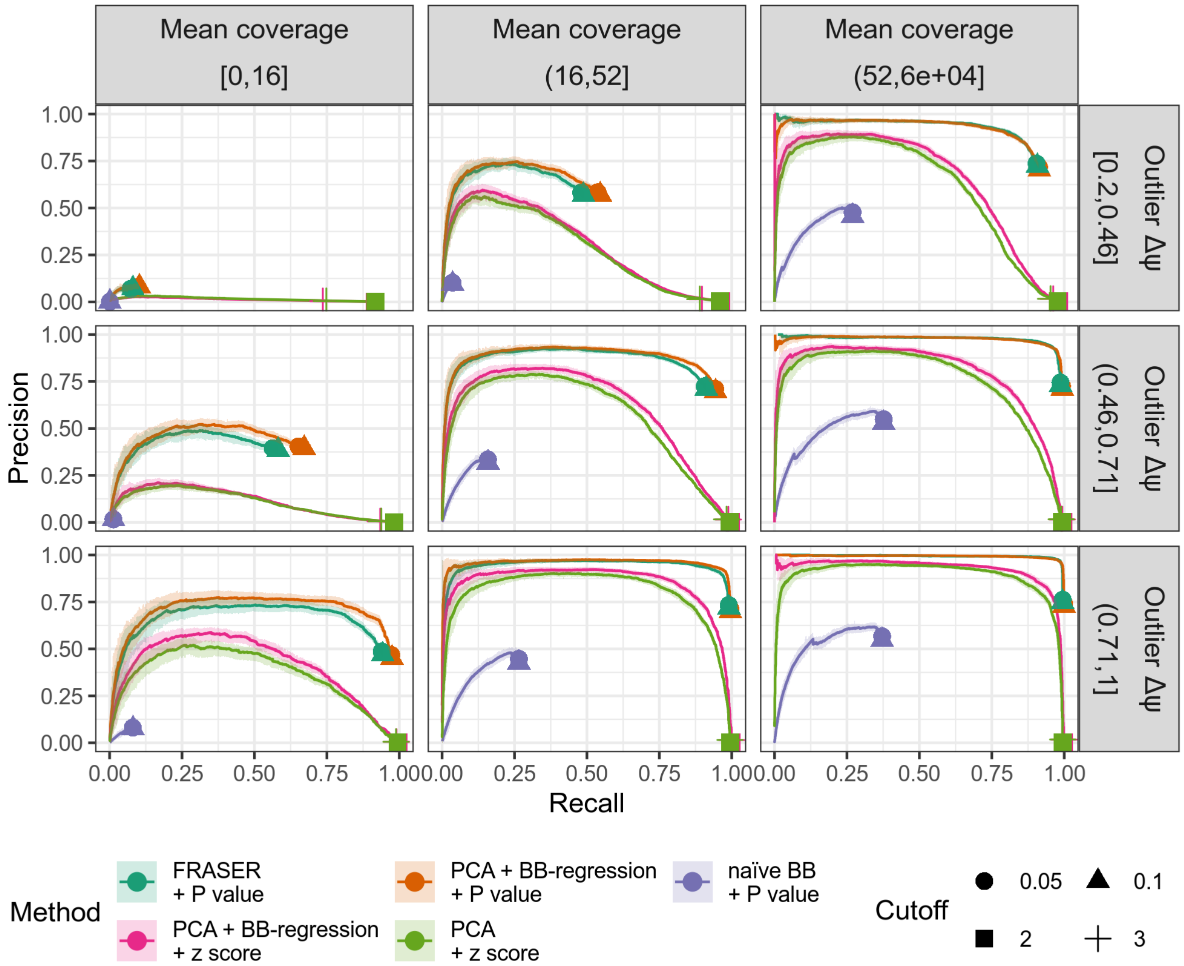

Figure S9: **Splicing outlier detection benchmark in GTEx for ψ_3.** The proportion of simulated outliers among reported outliers (precision, y-axis) plotted against the proportion of reported simulated outliers among all simulated outliers (recall, x-axis) for increasing beta-binomial P values computed using count ratio expectations based on FRASER (green), a beta-binomial regression on the latent space (orange), or on raw count ratios (purple, naïve BB) and for decreasing absolute z scores on top of a beta-binomial regression (pink) or PCA (light green). Additionally, all events with $|\Delta\psi| < 0.1$ are ranked last. Plots are stratified equally by injected amplitudes ($\Delta\psi$, by row) and junction coverage (by column). The points indicate commonly applied cutoffs (FDR < 0.1 and < 0.05 and absolute z scores > 2 and > 3). The darker lines mark the precision-recall curves computed for the full dataset while the light ribbons around the curves depict 95% confidence bands estimated by bootstrapping. Abbreviations: BB, beta-binomial; PCA, principal component analysis.

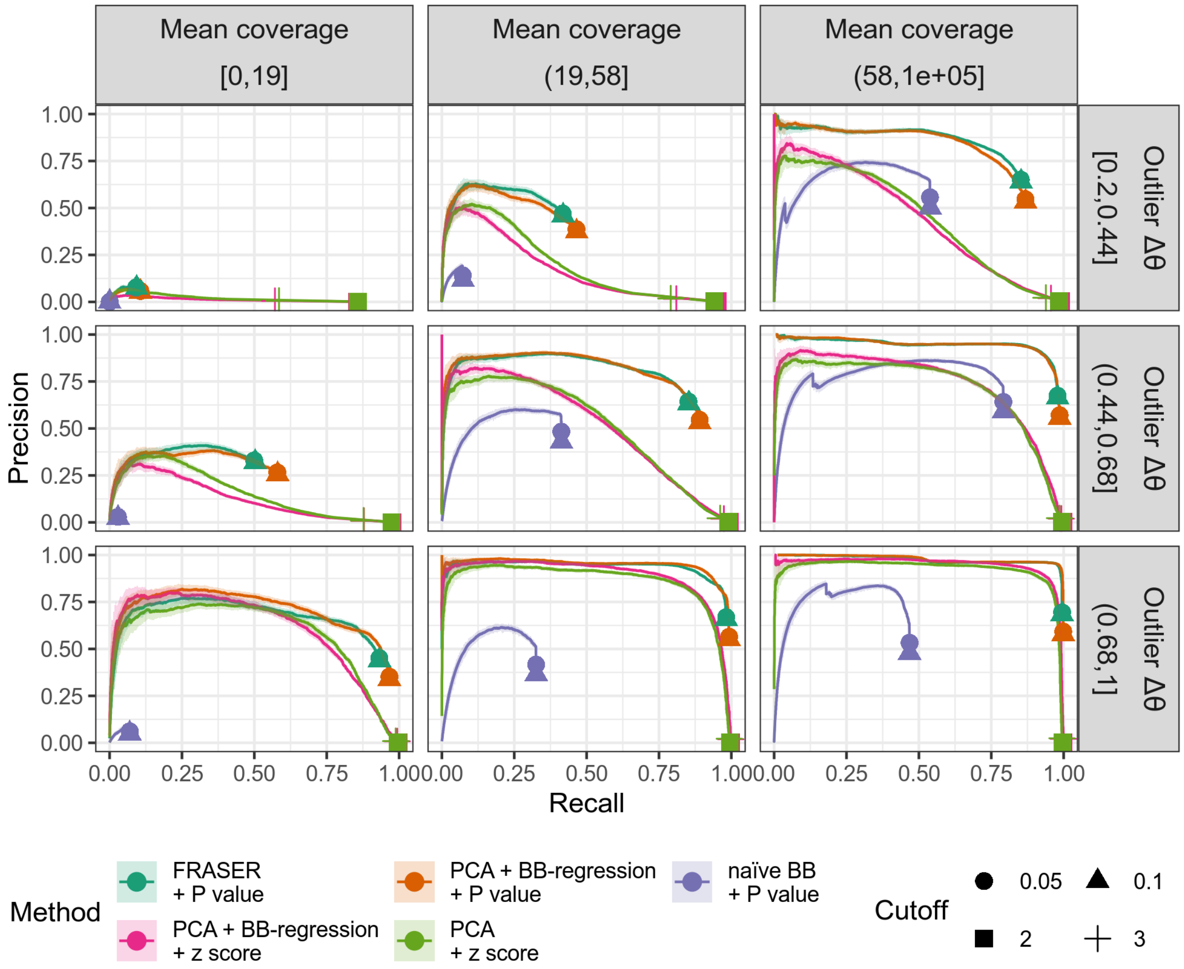

Figure S10: **Splicing outlier detection benchmark in GTEx for θ.** Same as Figure **S9**, but based on the splicing efficiency metric θ.

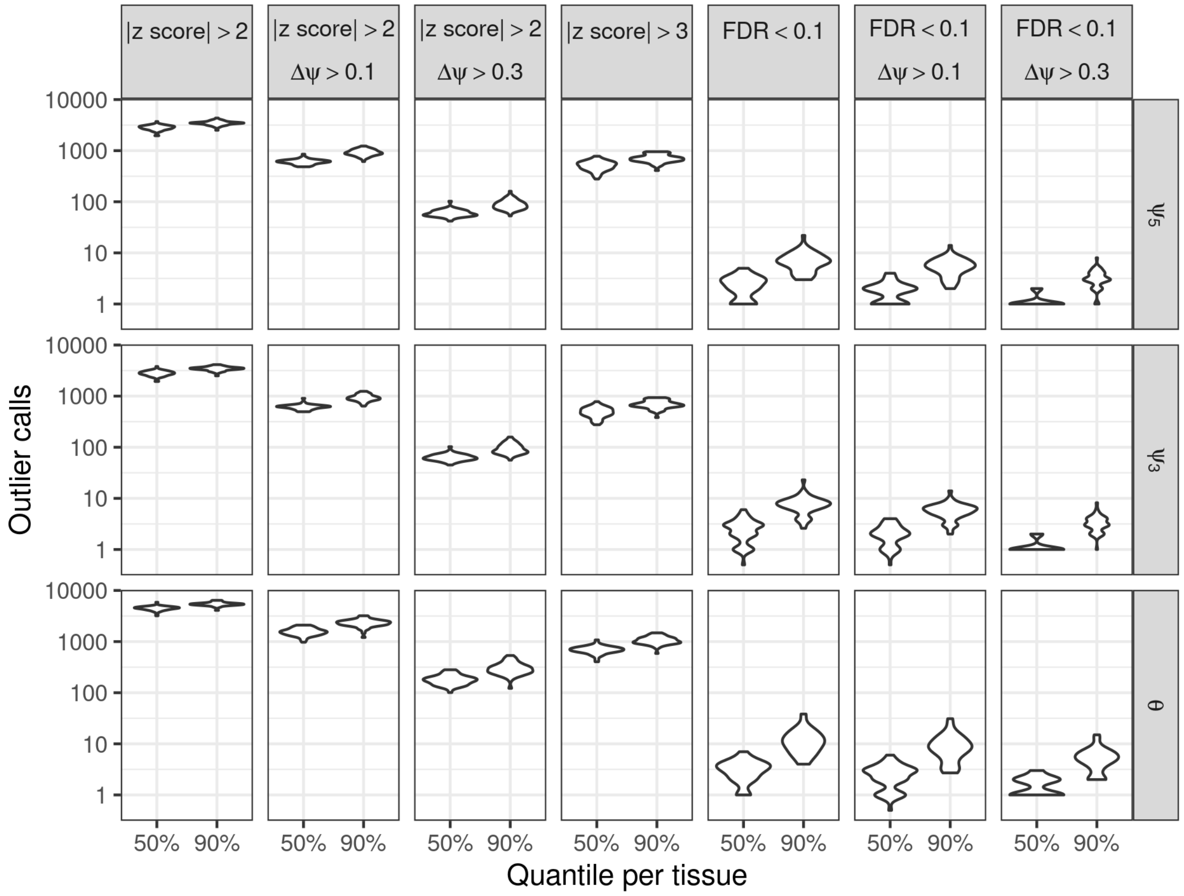

Figure S11: **Distribution of aberrant splicing events in GTEx.** Distribution of aberrant splicing events (y-axis) called using different cutoffs based on the FRASER normalization are plotted against two quantiles (rows, 50% and 90%) across samples in a tissue for all 48 GTEx tissues. The plot is stratified by the splicing metrics (rows) and commonly applied cutoffs (columns). Overall z score based cutoffs report in magnitudes more outlier than significant based and FDR controlled cutoff approaches.

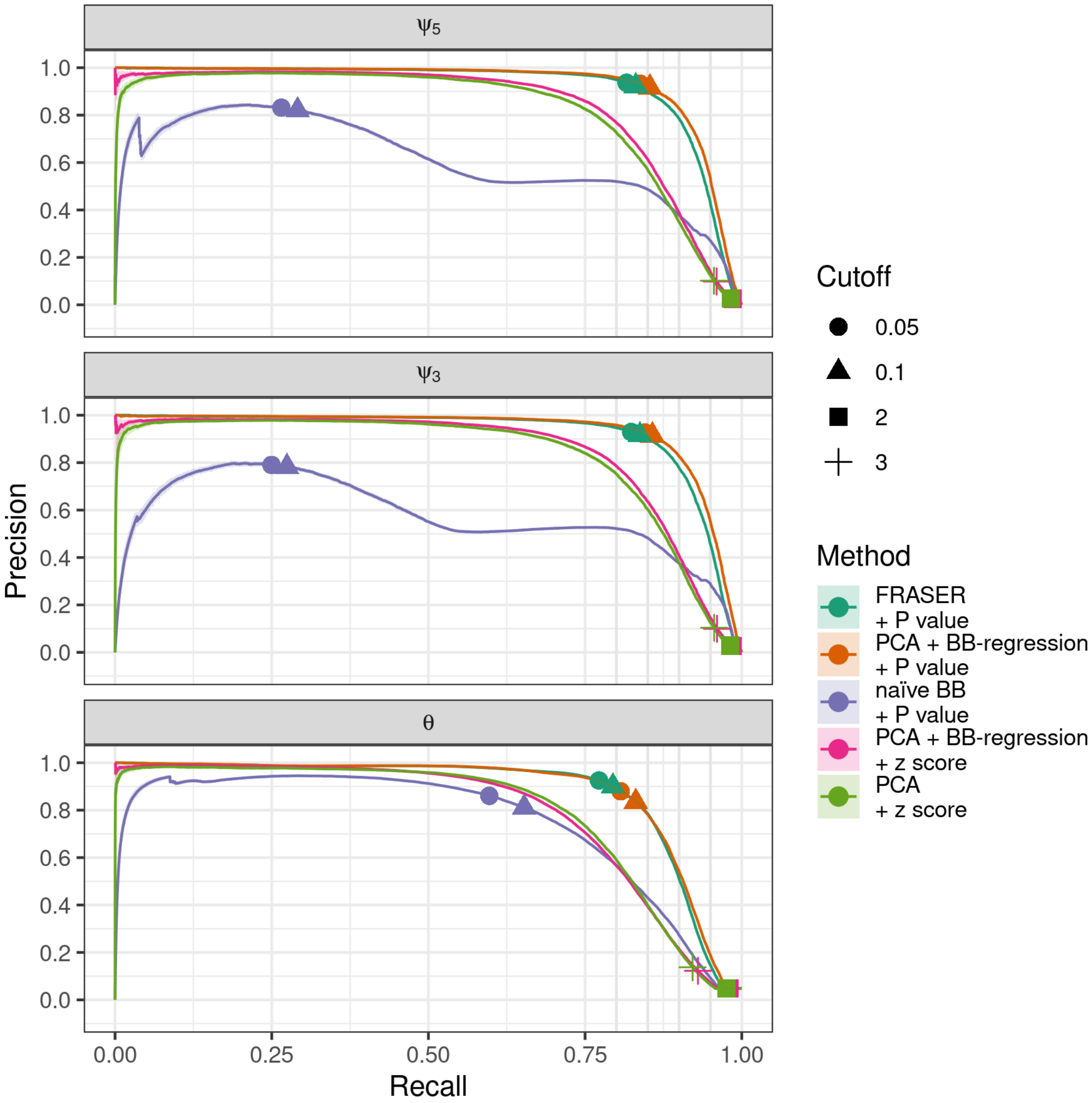

Figure S12: **Performance benchmark using artificially injected outliers.** The proportion of simulated outliers among reported outliers (precision, y-axis) plotted against the proportion of reported simulated outliers among all simulated outliers (recall, x-axis) for increasing beta-binomial P values computed using count ratio expectations based on FRASER (green), a beta-binomial regression on the latent space (orange), or on raw count ratios (purple, naïve BB) and for decreasing absolute z scores on top of a beta-binomial regression (pink) or PCA (light green). The data is stratified by the different splice metrics: ψ_5, ψ_3, and θ (rows). The points indicate commonly applied cutoffs (FDR < 0.1 and < 0.05 and absolute z scores > 2 and > 3). The darker lines mark the precision-recall curves computed for the full dataset while the light ribbons around the curves depict 95% confidence bands estimated by bootstrapping. Abbreviations: BB, beta-binomial; PCA, principal component analysis.

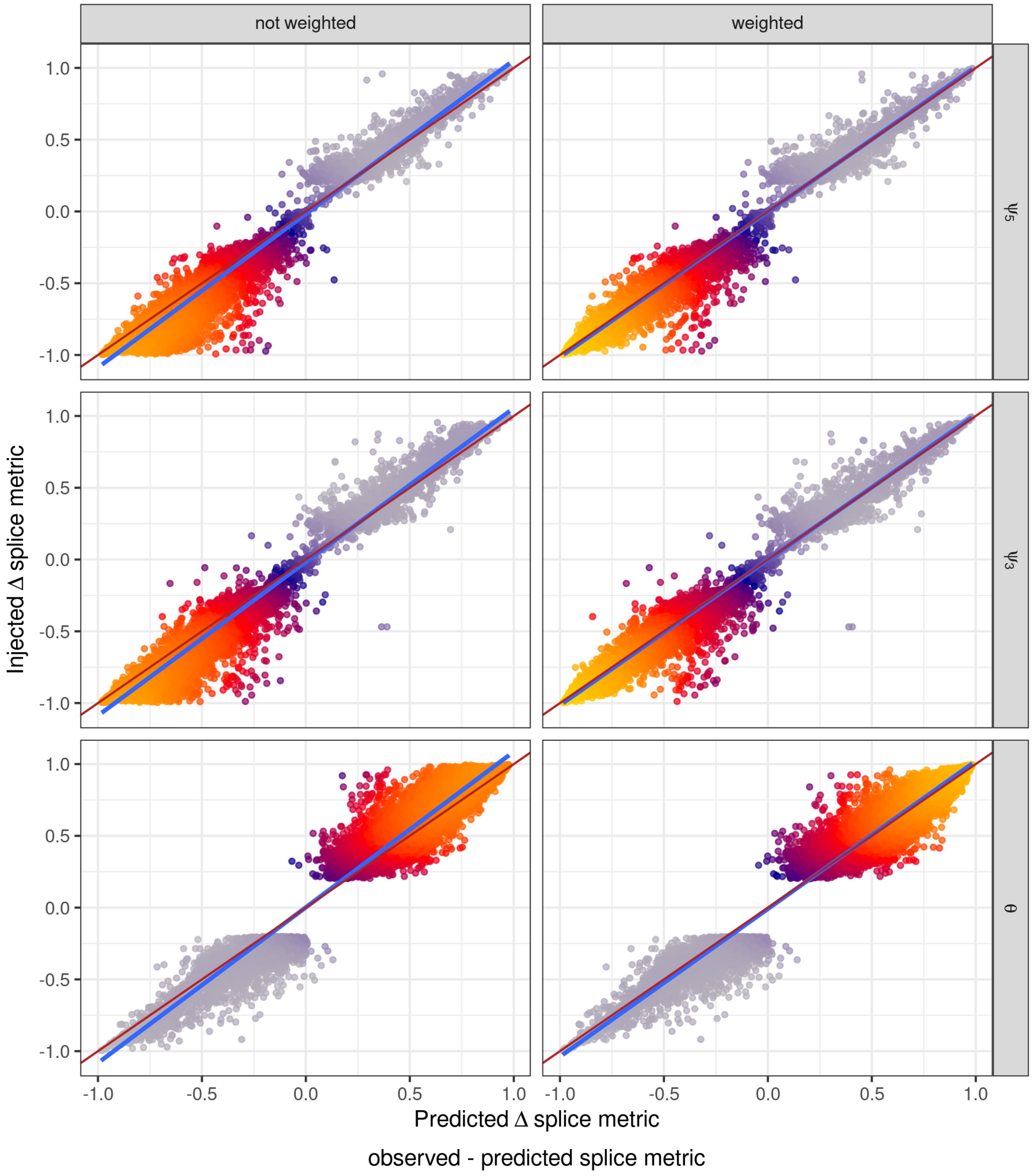

Figure S13: **Using a weighted beta-binomial loss for robustness against outlier data points.** For each injected outlier data point, the effect size of the injection (y-axis, $\Delta\bullet$) is plotted against the predicted difference (x-axis, observed $\Delta\bullet$ − predicted $\Delta\bullet$) based on the beta-binomial regression fit. The plot is stratified by the three splicing metrics ψ_5, ψ_3, and θ (rows) and by loss function used in the beta-binomial regression (columns, non weighted and weighted). The blue line corresponds to a linear regression. The red line indicates the diagonal, which would be the perfect fit. The data is based on the suprapubic skin GTEx tissue.

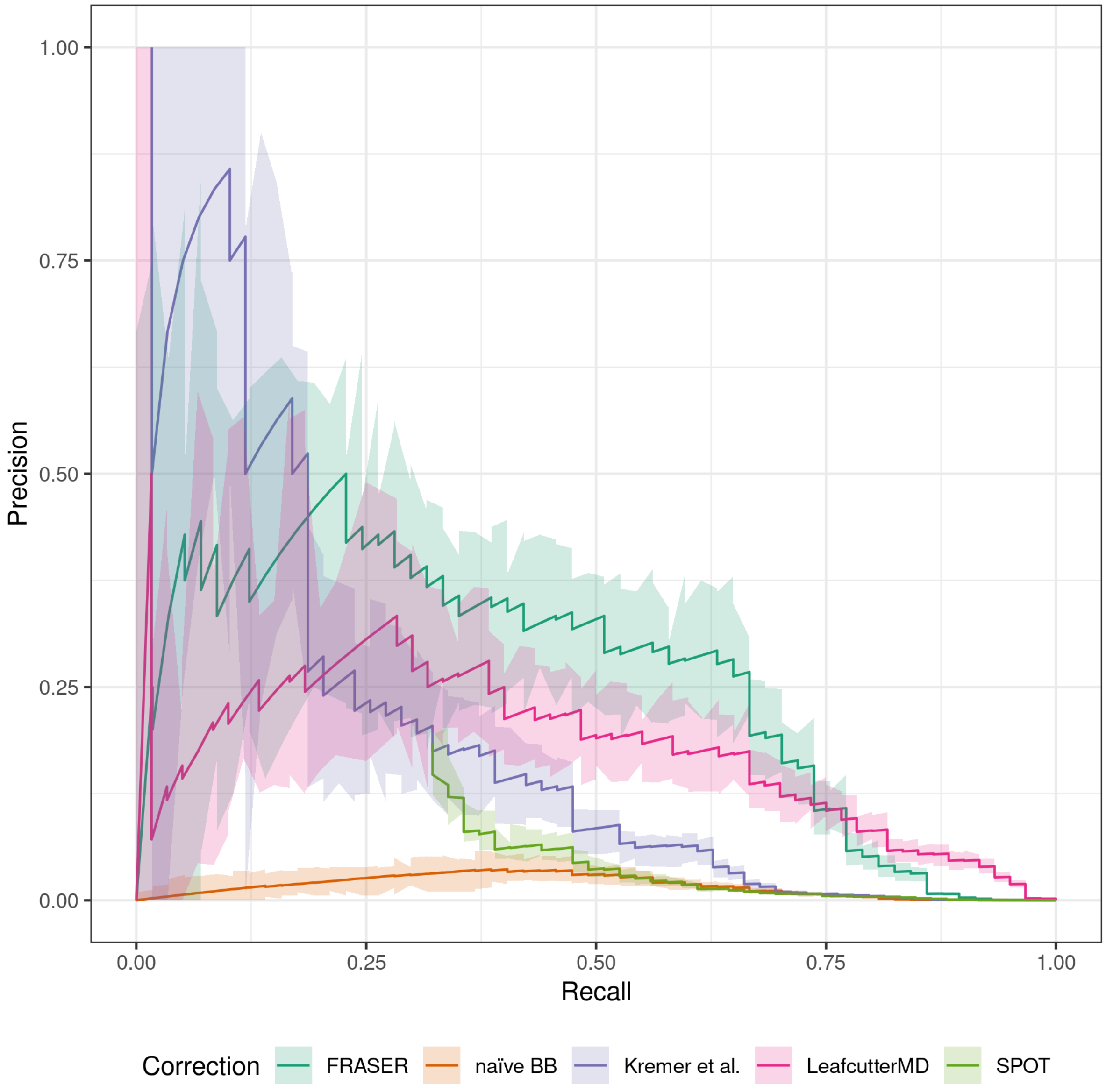

Figure S14: **Recall analysis of injected outliers by interchanging read counts of alternatively spliced genes between tissues.** Proportion of simulated outliers among reported outliers (precision, y-axis) against the proportion of reported simulated outliers among all simulated outliers (recall, x-axis) for increasing beta-binomial P values computed using count ratio expectations based on FRASER (green) and on raw count ratios (orange, naïve BB) and Dirichlet-Multinomial P values computed using the method from Kremer et al. (purple), LeafcutterMD (pink), and SPOT (light green). The darker lines mark the precision-recall curves computed for the full dataset while the light ribbons around the curves depict 95% confidence bands estimated by bootstrapping. Abbreviations: BB, beta-binomial.

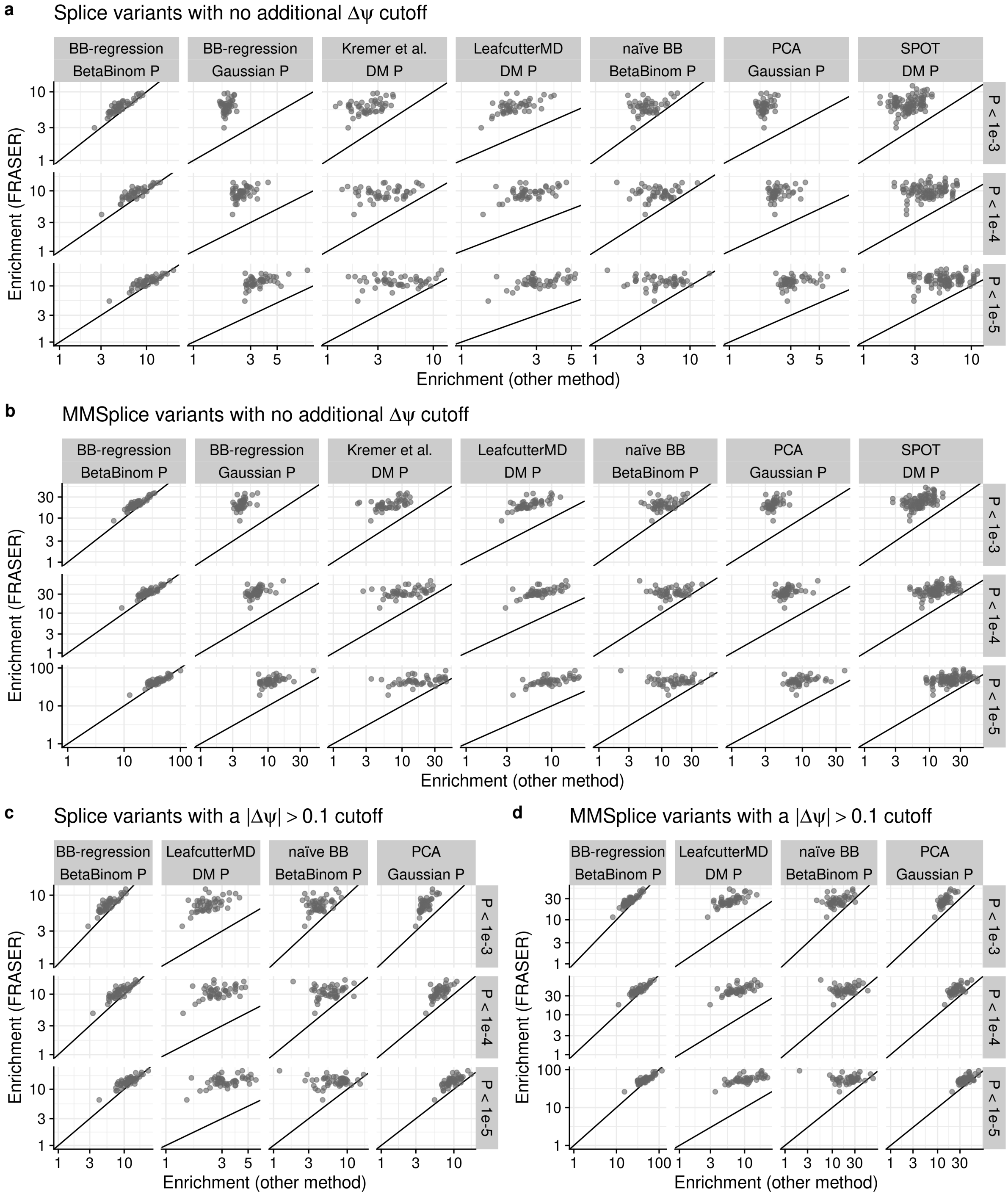

Figure S15: **Gene-based rare variant enrichment analysis.** **(a)** Enrichment using FRASER (y-axis) against enrichment using different aberrant splicing detection methods (x-axis, columns) for rare variants located in a splice region. The method applied are a beta-binomial regression on the latent space with (i) beta-binomial P values and (ii) Gaussian P values, Dirichlet-Multinomial P values based on (iii) Kremer et al. and (iv) LeafcutterMD, (v) naïve beta-binomial P values, (vi) PCA-based Gaussian P values, and Dirichlet-Multinomial P values based on (vii) SPOT. The enrichment is calculated for different nominal P value cutoffs (rows). Each dot represents a GTEx tissue (n=48). **(b)** The same as (a) but based on rare variants predicted to affect splicing by MMSplice. **(c)** The same as (a) but an additional $|\Delta\psi| > 0.1$ cutoff was applied on the aberrant splicing calls. **(d)** The same as b but an additional $|\Delta\psi| > 0.1$ cutoff was applied on the aberrant splicing calls. Abbreviations: BB, beta-binomial; BetaBinom, beta-binomial; DM, dirichlet-multinomial.

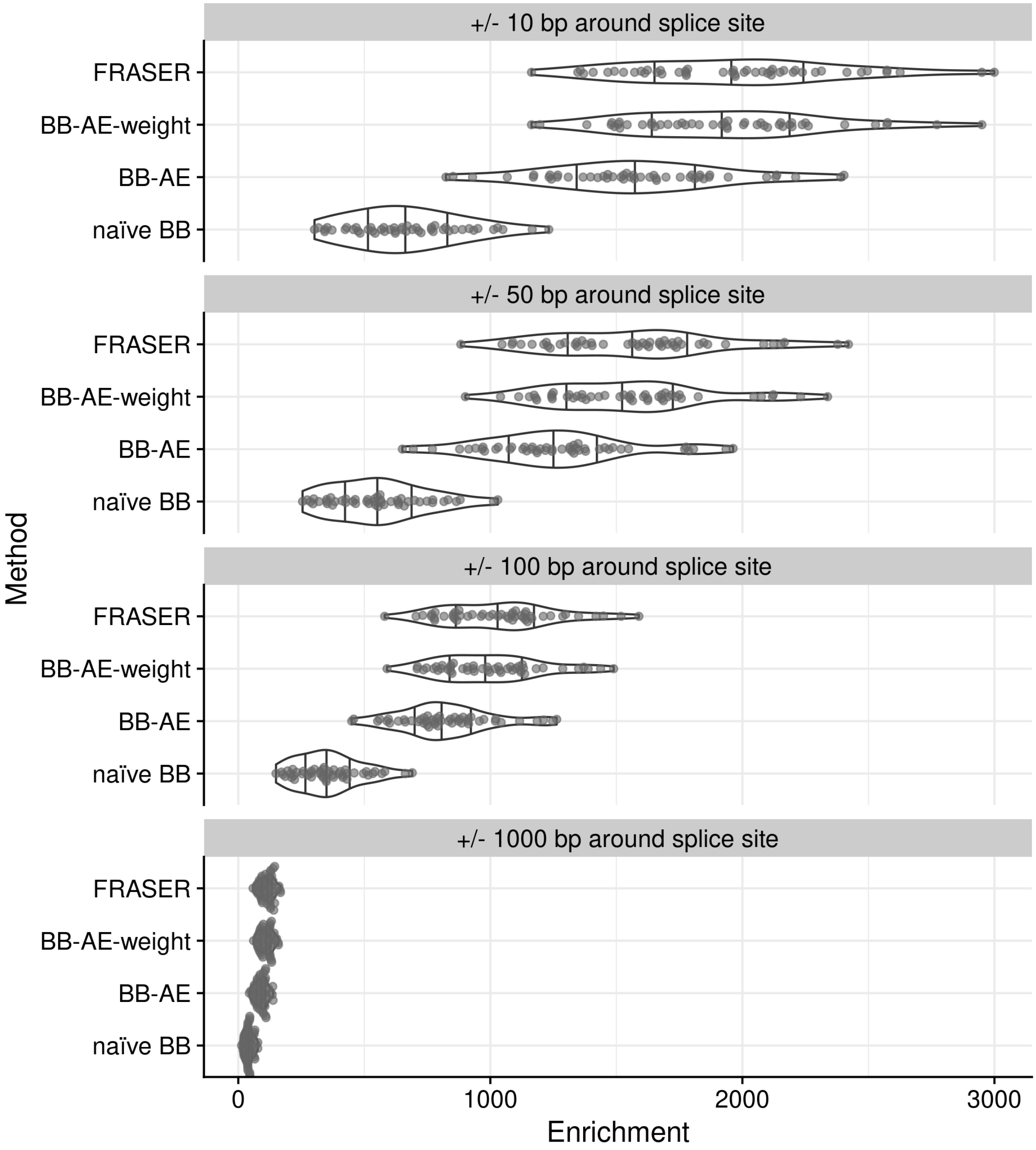

Figure S16: **Splice-site-based rare variant enrichment analysis.** Violin plots of splice-site-based rare MMSplice variant enrichments (x-axis) for different correction methods (y-axis) and various variant range cutoffs (facets). The methods are FRASER, beta-binomial autoencoder with a weighted loss (BB-AE-weight), beta-binomial autoencoder (BB-AE), and no covariation correction (naïve BB). Each dot represents a GTEx tissue (n=48). The violin represents the density of the data points and the middle line indicates the median while the first and last line indicate the first and third quartiles, respectively.

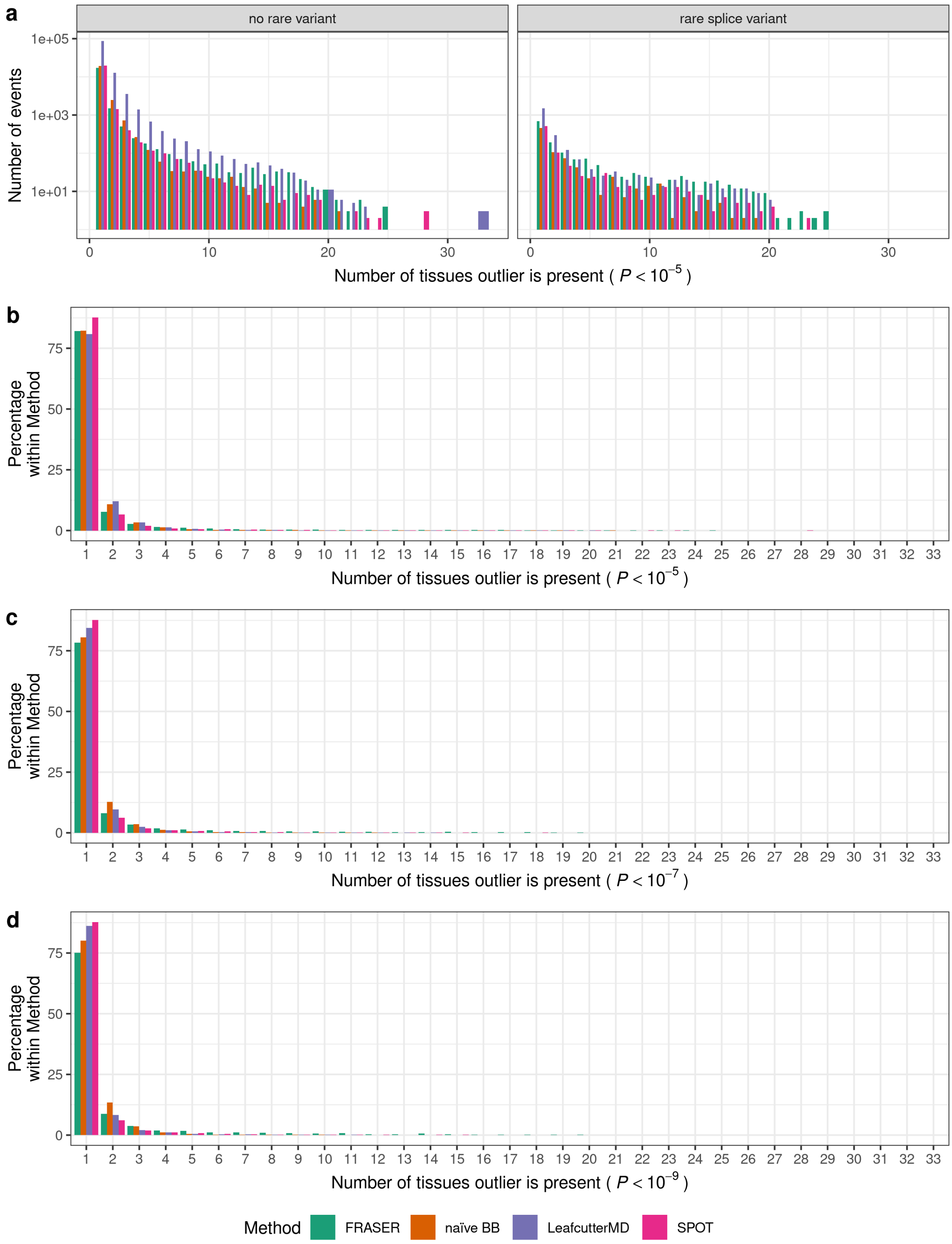

Figure S17: **Reproducibility of splicing outlier calls across GTEx tissues. (a)** Barplot of the number of gene-level events (y-axis) against their reproducibility (x-axis) across GTEx tissues. The reproducibility is defined as the number of tissues an event is observed at a nominal P value $p < 10^{-3}$ given it was observerd at least once at a nominal P value $p < 10^{-5}$. The data is stratified by associated variant status and grouped by the different methods: FRASER (green), naïve beta-binomial (orange), LeafcutterMD (purple), and SPOT (pink). **(b)** Same as (a) but plotted as the proportion (y-axis) of reproducible gene-level splicing outlier calls in GTEx tissues (number of tissues, x-axis). **(c)** Same as (b) but with at least one call at a nominal P value $p < 10^{-7}$. **(d)** Same as (b) but with at least one call at a nominal P value $p < 10^{-9}$. Abbr.: BB, beta-binomial.

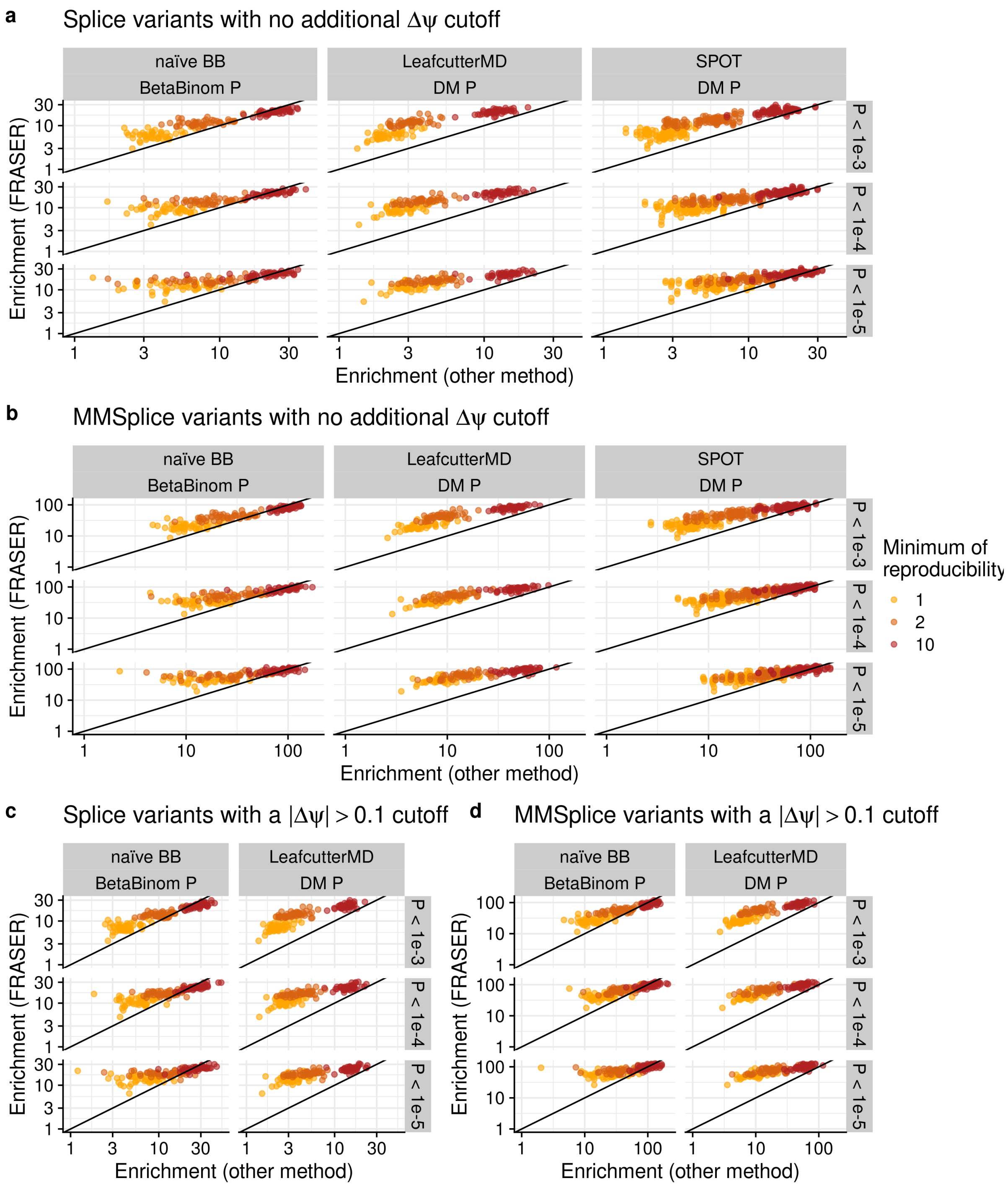

Figure S18: **Gene-based rare variant enrichment on reproducible splicing outlier calls.** **(a)** Enrichment using FRASER (y-axis) against enrichment using different aberrant splicing detection methods (x-axis, columns) for rare variants located in a splice region. The methods applied are (i) naïve beta-binomial P values, Dirichlet-Multinomial P values based on (ii) LeafcutterMD and (iii) SPOT. The enrichment is calculated for different nominal P value cutoffs (rows) and increased reproducibility cutoff: $p < 10^{-3}$ in at least 0, 1, or 9 other tissues (color). Each dot represents a GTEx tissue. **(b)** The same as (a) but based on rare variants predicted to affect splicing by MMSplice. **(c)** The same as (a) but an additional $|\Delta\psi| > 0.1$ cutoff was applied on the aberrant splicing calls. **(d)** The same as b but an additional $|\Delta\psi| > 0.1$ cutoff was applied on the aberrant splicing calls. Abbreviations: BB, beta-binomial; BetaBinom, beta-binomial; DM, dirichlet-multinomial.

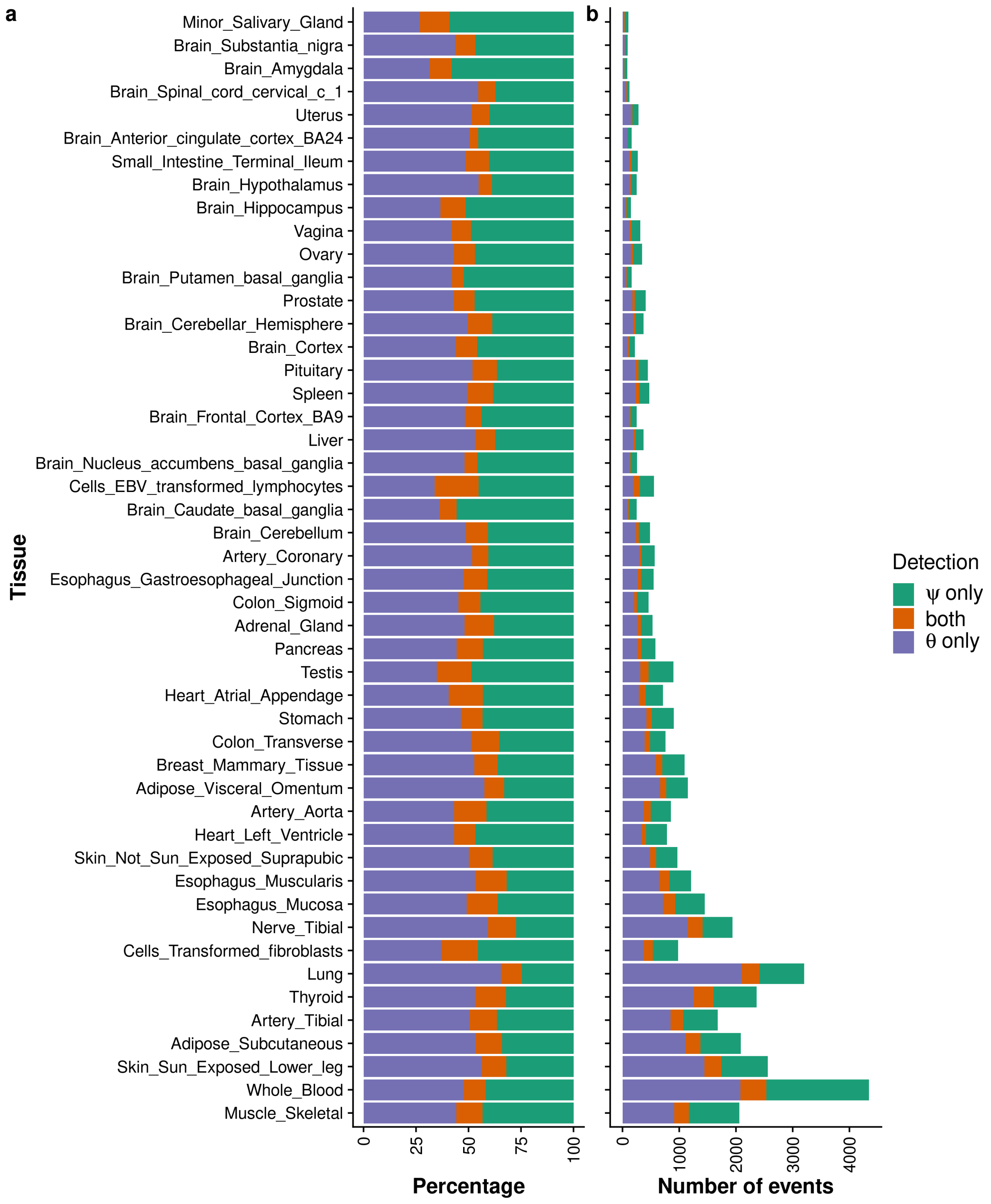

Figure S19: **Contribution of intron retention in aberrant splicing.** **(a)** Barplot of the percentage of aberrant splicing events on the gene level grouped by the detection metrics: alternative splicing only (green, ψ metric), intron retention (purple, θ metric), and both (red). **(b)** Same as (a) but with the absolute number of detected events.

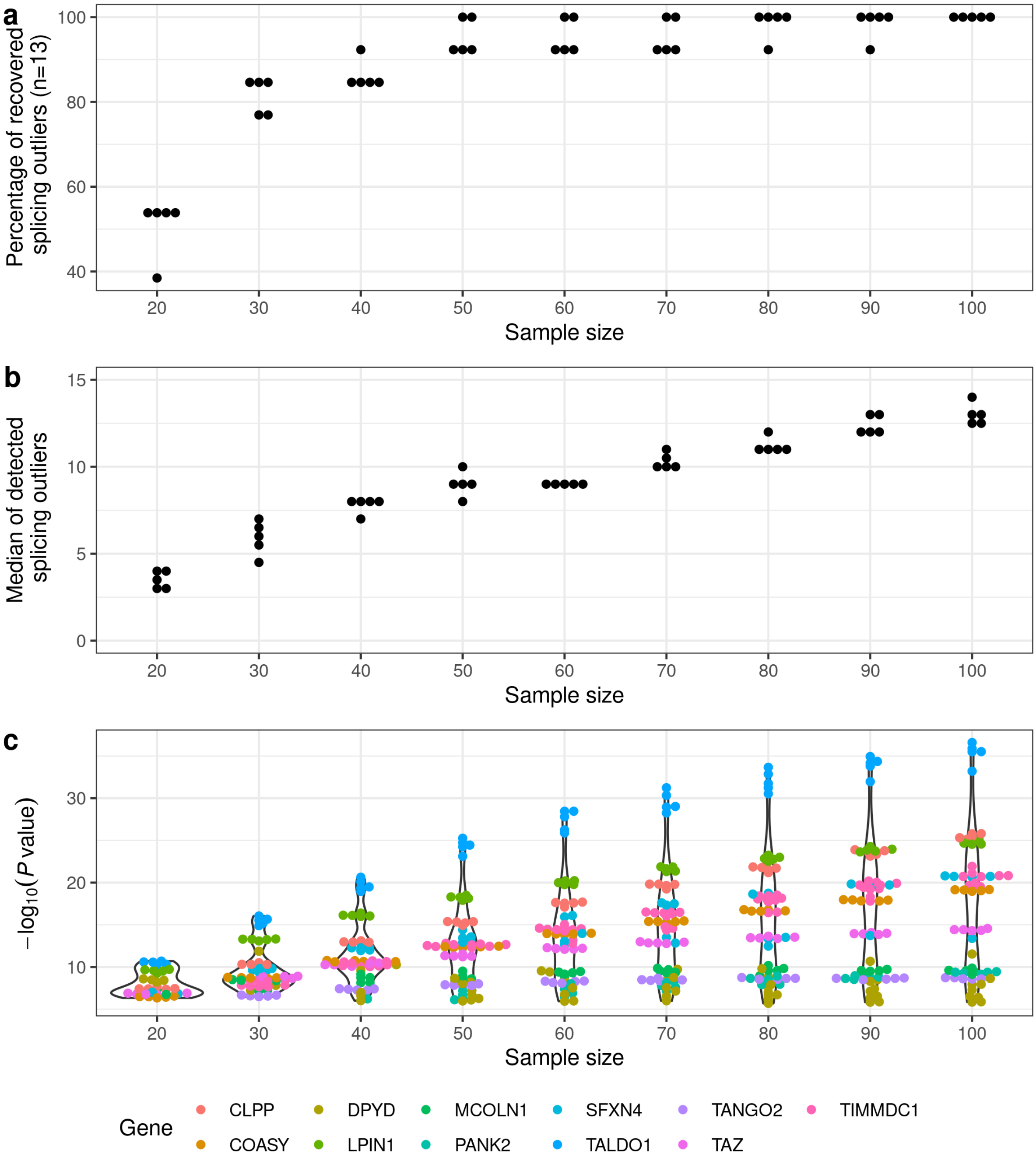

Figure S20: **Sample size analysis in Kremer et al. (a)** The percentage of the recovered known disease-causing splicing outliers in Kremer et al. (y-axis) is plotted against the used sample size (x-axis). The sample selection was repeated 5 times (dots). **(b)** The median of splicing outliers across all samples (y-axis) is plotted against the used sample size (x-axis). **(c)** The negative $\log_{10}$ P-value for all known disease-causing splicing outliers (y-axis) is plotted against the used sample size (x-axis). The color depicts the gene with a known splice defect. The violin depicts the density of the data points. In panel (c), P values were calculated two-sided with the beta-binomial distribution. To obtain the outlier calls in panels (a,b), P values were corrected for multiple comparisions (Methods).

Supplementary Note 1: Splice-site-based rare MM-Splice variant enrichment

For the local splice site enrichment, we used the same MMSplice variant set as described in the main Method section. We then computed enrichments for rare splicing variants found within a given region around an aberrently spliced donor or acceptor as the proportion of outlier splice sites having a rare splicing variant over the proportion of non-outlier splice sites having a rare splicing variant. A splice site (donor or acceptor) was called aberrantly spliced, if any of the three metrics (ψ_5, ψ_3, and θ) called it aberrant. Variants where associated with a splice site, if it was within 10, 50, 100, and 1000 base pairs down- and upstream.

Supplementary Note 2: Natural Z Score Calculation

The natural z scores z_{ij}^n are calculated per intron on the difference between the measured ψ value including pseudocounts and the proportion expectation μ_{ij}, as follows:

$$\begin{aligned} z_{ij}^n &= \frac{\Delta\psi_{ij} - \overline{\Delta\psi_j}}{\mathrm{sd}(\Delta\psi_j)} \\ \Delta\psi_{ij} &= \psi_{ij} - \mu_{ij} \\ &= \frac{k_{ij} + 1}{n_{ij} + 2} - \mu_{ij}. \end{aligned}$$

Supplementary Note 3: Fitting of the parameters

All notations are introduced in the Materials and Methods section.

Beta-binomial model

We use the following parameterization of the beta-binomial distribution:

$$P(k|n, \alpha, \beta) = \frac{\Gamma(n+1)}{\Gamma(k+1)\Gamma(n-k+1)} \frac{\Gamma(\alpha+k)\Gamma(\beta+n-k)}{\Gamma(\alpha+\beta+n)} \frac{\Gamma(\alpha+\beta)}{\Gamma(\alpha)\Gamma(\beta)},$$

where

$$\alpha = \mu \left(\frac{1-\rho}{\rho}\right) \text{ and } \beta = (\mu - 1)\left(\frac{\rho - 1}{\rho}\right).$$

The variance of a beta-binomially distributed variable X is given by:

$$\mathrm{Var}(X) = n\mu(1-\mu)(1 + (n-1)\rho)$$

and the variance of the count ratio $\frac{k}{n}$ is then

$$\mathrm{Var}\left(\frac{X}{n}\right) = \frac{\mu(1-\mu)(1+(n-1)\rho)}{n}.$$

The negative log-likelihood of the beta-binomial distribution

The negative log-likelihood (nll) of the model is given by:

$$\begin{aligned}\mathrm{nll} = &-\sum_{ij}\log(\Gamma(n_{ij}+1)) + \sum_{ij} log(\Gamma(k_{ij}+1)) + \sum_{ij}\log(\Gamma(n_{ij}-k_{ij}+1)) \\ &-\sum_{ij}\log(\Gamma(\alpha_{ij}+k_{ij})) - \sum_{ij}\log(\Gamma(\beta_{ij}+n_{ij}-k_{ij})) + \sum_{ij}\log(\Gamma(\alpha_{ij}+\beta_{ij}+n_{ij})) \\ &+\sum_{ij}\log(\Gamma(\alpha_{ij})) + \sum_{ij}\log(\Gamma(\beta_{ij})) - \sum_{ij}\log(\Gamma(\alpha_{ij}+\beta_{ij})).\end{aligned}$$

Fitting the intra-class correlation parameter ρ

For the optimization of the intra-class correlation parameter ρ only the ρ depending terms in nll need to be considered and yield the following truncated form of the negative log likelihood, with pseudocounts of 1 and 2 added to k and n, respectively:

$$\begin{aligned}\mathrm{nll}_\rho = &-\sum_{ij}\log(\Gamma(\alpha_{ij}+k_{ij}+1)) - \sum_{ij}\log(\Gamma(\beta_{ij}+n_{ij}-k_{ij}+1)) + \sum_{ij}\log(\Gamma(\alpha_{ij}+\beta_{ij}+n_{ij}+2)) \\ &+\sum_{ij}\log(\Gamma(\alpha_{ij})) + \sum_{ij}\log(\Gamma(\beta_{ij})) - \sum_{ij}\log(\Gamma(\alpha_{ij}+\beta_{ij})).\end{aligned}$$

In the following y_{ij} is an element of $\mathbf{Y}$ defined as:

$$\mathbf{Y} = \widetilde{\mathbf{X}}\mathbf{W}_e\mathbf{W}_d + \mathbf{b}, \tag{1}$$

where the element $\widetilde{x}_{ij}$ of the matrix $\widetilde{\mathbf{X}}$ is given by:

$$\begin{aligned}\widetilde{x}_{ij} &= x_{ij} - \bar{x}_j, \\ x_{ij} &= \mathrm{logit}\left(\frac{k_{ij}+1}{n_{ij}+2}\right), \\ \mathrm{logit}(a) &= \log\frac{a}{1-a}.\end{aligned}$$

The expectations μ_{ij} are then modelled by:

$$\mu_{ij} = \sigma\left(y_{ij}\right) = \frac{e^{y_{ij}}}{1+e^{y_{ij}}}$$

Hence, nll_ρ can be rewritten as:

$$\begin{aligned}\text{nll}_\rho = &-\sum_{ij}\log\left(\Gamma\left(\frac{e^{y_{ij}}}{1+e^{y_{ij}}}\frac{1-\rho_{ij}}{\rho_{ij}}+k_{ij}+1\right)\right)\\ &-\sum_{ij}\log\left(\Gamma\left(\left(\frac{e^{y_{ij}}}{1+e^{y_{ij}}}-1\right)\frac{\rho_{ij}-1}{\rho_{ij}}+n_{ij}-k_{ij}+1\right)\right)\\ &+\sum_{ij}\log\left(\Gamma\left(\frac{1-\rho_{ij}}{\rho_{ij}}+n_{ij}+2\right)\right)\\ &+\sum_{ij}\log\left(\Gamma\left(\frac{e^{y_{ij}}}{1+e^{y_{ij}}}\frac{1-\rho_{ij}}{\rho_{ij}}\right)\right)\\ &+\sum_{ij}\log\left(\Gamma\left(\left(\frac{e^{y_{ij}}}{1+e^{y_{ij}}}-1\right)\frac{\rho_{ij}-1}{\rho_{ij}}\right)\right)\\ &-\sum_{ij}\log\left(\Gamma\left(\frac{1-\rho_{ij}}{\rho_{ij}}\right)\right)\end{aligned}$$

To fit ρ, we use *optimize* provided in the R base package *stats*. To not run into convergence issues or numerical instability of the log gamma function, we estimate the value of the log gamma function if not $-35 < y_{ij} < 30$.

Alternative approaches to model the encoder and decoder

For the optimization of the autoencoder model only the terms of nll that are dependent on $\mathbf{W}_e$ or $\mathbf{W}_d$ need to be considered. Since

$$\alpha + \beta = \frac{1-\rho}{\rho}$$

is independent of μ and therefore independent of $\mathbf{W}_e$ and $\mathbf{W}_d$, we do not have to consider the terms containing $\alpha + \beta$ and yield the following truncated form of the negative log likelihood, with pseudocounts of 1 and 2 added to k and n, respectively:

$$\text{nll}_{\mathbf{W}} = \sum_{ij}\log(\Gamma(\alpha_{ij})) + \sum_{ij}\log(\Gamma(\beta_{ij})) \tag{2}$$

$$-\sum_{ij}\log(\Gamma(\alpha_{ij}+k_{ij}+1)) - \sum_{ij}\log(\Gamma(\beta_{ij}+n_{ij}-k_{ij}+1)). \tag{3}$$

Hence, $\text{nll}_{\mathbf{W}}$ can be rewritten as:

$$
\begin{aligned}
\text{nll}_{\mathbf{W}} =& \sum_{ij} \log\left(\Gamma\left(\frac{e^{y_{ij}}}{1+e^{y_{ij}}}\frac{1-\rho_{ij}}{\rho_{ij}}\right)\right) \\
&+ \sum_{ij} \log\left(\Gamma\left(\left(\frac{e^{y_{ij}}}{1+e^{y_{ij}}}-1\right)\frac{\rho_{ij}-1}{\rho_{ij}}\right)\right) \\
&- \sum_{ij} \log\left(\Gamma\left(\frac{e^{y_{ij}}}{1+e^{y_{ij}}}\frac{1-\rho_{ij}}{\rho_{ij}} + k_{ij} + 1\right)\right) \\
&- \sum_{ij} \log\left(\Gamma\left(\left(\frac{e^{y_{ij}}}{1+e^{y_{ij}}}-1\right)\frac{\rho_{ij}-1}{\rho_{ij}} + n_{ij} - k_{ij} + 1\right)\right)
\end{aligned}
$$

We use L-BFGS[1] as implemented in *optim* to fit the autoencoder model as described in Methods.

Update of the encoder and decoder matrix

The updating of the matrix $\mathbf{W}_d$ is performed intron-wise whereas the encoder matrix $\mathbf{W}_e$ is performed on the full matrix. For each update step, the intron-wise or matrix-wise average negative log likelihood is minimized. To not run into convergence issues or numerical instability of the digamma function, we estimate the value of the digamma function ψ if not $-35 < y_{ij} < 30$. From Equation 1 and Equation 2, we obtain the gradients:

$$
\begin{aligned}
\frac{d\text{nll}}{d\mathbf{W}_e} &= \widetilde{\mathbf{X}}^T\mathbf{A}\mathbf{W}_d + \widetilde{\mathbf{X}}^T\mathbf{B}\mathbf{W}_d - \widetilde{\mathbf{X}}^T\mathbf{C}\mathbf{W}_d - \widetilde{\mathbf{X}}^T\mathbf{D}\mathbf{W}_d \\
\frac{d\text{nll}}{d\mathbf{W}_d} &= \mathbf{A}^T\widetilde{\mathbf{X}}\mathbf{W}_e + \mathbf{B}^T\widetilde{\mathbf{X}}\mathbf{W}_e - \mathbf{C}^T\widetilde{\mathbf{X}}\mathbf{W}_e - \mathbf{D}^T\widetilde{\mathbf{X}}\mathbf{W}_e \\
\frac{d\text{nll}}{db_j} &= \sum_i a_{ij} + b_{ij} - c_{ij} - d_{ij}
\end{aligned}
$$

,

where the components of the matrices $\mathbf{A}, \mathbf{B}, \mathbf{C}$ and $\mathbf{D}$ are computed by:

$$
\begin{aligned}
a_{ij} &= \psi\left(\frac{e^{y_{ij}}}{1+e^{y_{ij}}}\cdot r_{ij}\right)\cdot r_{ij}\cdot v_{ij} \\
b_{ij} &= \psi\left(\left(\frac{e^{y_{ij}}}{1+e^{y_{ij}}}-1\right)\cdot(-r_{ij}\right)\cdot(-r_{ij})\cdot v_{ij} \\
c_{ij} &= \psi\left(\frac{e^{y_{ij}}}{1+e^{y_{ij}}}\cdot r_{ij} + k_{ij} + 1\right)\cdot r_{ij}\cdot v_{ij} \\
d_{ij} &= \psi\left(\left(\frac{e^{y_{ij}}}{1+e^{y_{ij}}}-1\right)\cdot(-r_{ij} + n_{ij} - k_{ij} + 1\right)\cdot(-r_{ij})\cdot v_{ij} \\
v_{ij} &= \frac{e^{y_{ij}}}{(1+e^{y_{ij}})^2} \\
r_{ij} &= \frac{1-\rho_j}{\rho_j}
\end{aligned}
$$

.

References

[1] Byrd, R., Lu, P., Nocedal, J., and Zhu, C. (1995). A Limited Memory Algorithm for Bound Constrained Optimization. SIAM Journal on Scientific Computing *16*, 1190–1208.
