# Supplementary material for: Detection of aberrant splicing events in RNA-seq data using FRASER
Source: Nat Commun. 2021 Jan 22;12:529. doi: 10.1038/s41467-020-20573-7 (PMC7822922; doi:10.1038/s41467-020-20573-7)
Supplement: Supplementary file 3 — Reporting Summary [file 41467_2020_20573_MOESM3_ESM.pdf]

nature research

Corresponding author(s): Julien Gagneur

Last updated by author(s): Nov 10, 2020

Reporting Summary

Nature Research wishes to improve the reproducibility of the work that we publish. This form provides structure for consistency and transparency in reporting. For further information on Nature Research policies, see our Editorial Policies and the Editorial Policy Checklist.

Statistics

For all statistical analyses, confirm that the following items are present in the figure legend, table legend, main text, or Methods section.

n/a	Confirmed	
☐	☒	The exact sample size (*n*) for each experimental group/condition, given as a discrete number and unit of measurement
☐	☒	A statement on whether measurements were taken from distinct samples or whether the same sample was measured repeatedly
☐	☒	The statistical test(s) used AND whether they are one- or two-sided<br>*Only common tests should be described solely by name; describe more complex techniques in the Methods section.*
☐	☒	A description of all covariates tested
☐	☒	A description of any assumptions or corrections, such as tests of normality and adjustment for multiple comparisons
☐	☒	A full description of the statistical parameters including central tendency (e.g. means) or other basic estimates (e.g. regression coefficient) AND variation (e.g. standard deviation) or associated estimates of uncertainty (e.g. confidence intervals)
☐	☒	For null hypothesis testing, the test statistic (e.g. *F*, *t*, *r*) with confidence intervals, effect sizes, degrees of freedom and *P* value noted<br>*Give P values as exact values whenever suitable.*
☒	☐	For Bayesian analysis, information on the choice of priors and Markov chain Monte Carlo settings
☐	☒	For hierarchical and complex designs, identification of the appropriate level for tests and full reporting of outcomes
☐	☒	Estimates of effect sizes (e.g. Cohen's *d*, Pearson's *r*), indicating how they were calculated

Our web collection on statistics for biologists contains articles on many of the points above.

Software and code

Policy information about availability of computer code

Data collection

We downloaded BAM and VCF files of the GTEx version V7 from dbGaP (phs000424.v7.p2).
The same dataset (RNA-seq BAM files) was used as in the Kremer et al. study (doi: 10.1038/ncomms15824).
No specific software was used to download or acquire the data.

Data analysis

The full analysis code for this study can be found on GitHub here:
https://github.com/gagneurlab/FRASER-analysis
The R package (FRASER) described in this manuscript and used throughout the study can be found on Bioconductor here:
http://bioconductor.org/packages/release/bioc/html/FRASER.html

Specifically we used the following software packages with the corresponding version or GitHub commit:

* R (3.6.1)
** Bioconductor (3.11)
** FRASER (1.3.0)
** Rsubread (2.2.6)
** pcaMethods (1.80.0)
** GenomicAlignments (1.24.0)
** GenomicRanges (1.40.0)
** VGAM (1.1-1)
** pheatmap (1.0.12)
** ggplot2 (3.2.1)
* Leafcutter/LeafcutterMD (https://github.com/davidaknowles/leafcutter @f330165)

* SPOT (https://github.com/BennyStrobes/SPOT @5f29563)
* MMSplice (https://github.com/gagneurlab/MMSplice_MTSplice @39cc426)
* STAR (2.4.2a)

For manuscripts utilizing custom algorithms or software that are central to the research but not yet described in published literature, software must be made available to editors and reviewers. We strongly encourage code deposition in a community repository (e.g. GitHub). See the Nature Research guidelines for submitting code & software for further information.

Data

Policy information about availability of data

All manuscripts must include a data availability statement. This statement should provide the following information, where applicable:

- Accession codes, unique identifiers, or web links for publicly available datasets
- A list of figures that have associated raw data
- A description of any restrictions on data availability

Split read data for the Kremer et al. dataset can be accessed through Zenodo: https://doi.org/10.5281/zenodo.4271599

Field-specific reporting

Please select the one below that is the best fit for your research. If you are not sure, read the appropriate sections before making your selection.

[x] Life sciences [ ] Behavioural & social sciences [ ] Ecological, evolutionary & environmental sciences

For a reference copy of the document with all sections, see nature.com/documents/nr-reporting-summary-flat.pdf

Life sciences study design

All studies must disclose on these points even when the disclosure is negative.

Sample size	We used 7,842 RNA-seq samples from 48 tissues of 543 individuals from the GTEx dataset (dbGaP: phs000424.v7.p2).<br>We used 119 RNA-seq samples from the Kremer et al. study (doi: 10.1038/ncomms15824).<br><br>For both datasets, we used all available samples/tissues within the dataset passing exclusion filters and number of samples per tissues (n >= 50). The minimum of 50 samples was chosen based on our power analysis (Supplemental Figure S20).
Data exclusions	We used the same rational on sample exclusion as done in the GTEx paper (doi: 10.1038/nature24277)<br>1) We filtered out samples with a low RIN number (RIN <= 5.7)<br>2) We excluded samples that were also excluded by the GTEx paper<br><br>In addition, we excluded any replicated sample to match experiment setups in rare disease diagnostics. Finally, we considered only tissues with at least 50 samples after filtering to meet our power analysis threshold (Supplemental Figure S20).
Replication	Not applicable. We did replicate our findings in silico (e.g. extensive benchmarks, bootstrapping), since we did not generate any experimental data.
Randomization	No randomization was performed. The sample covariation was corrected using a denoising autoencoder scheme. The optimal dimension for the denoising autoencoder was selected as to maximize the area under the precision-recall curve for identifying the corrupted read ratios.
Blinding	No new wet lab experiments were performed. Hence, blinding does not apply on the data generation side.<br><br>On the algorithmic side, blinding was ensured by injecting outliers in datasets and letting outlier detection algorithms run without any knowledge of their identity.

Reporting for specific materials, systems and methods

We require information from authors about some types of materials, experimental systems and methods used in many studies. Here, indicate whether each material, system or method listed is relevant to your study. If you are not sure if a list item applies to your research, read the appropriate section before selecting a response.

Materials & experimental systems

n/a	Involved in the study
☒	☐ Antibodies
☒	☐ Eukaryotic cell lines
☒	☐ Palaeontology and archaeology
☒	☐ Animals and other organisms
☒	☐ Human research participants
☒	☐ Clinical data
☒	☐ Dual use research of concern

Methods

n/a	Involved in the study
☒	☐ ChIP-seq
☒	☐ Flow cytometry
☒	☐ MRI-based neuroimaging
